# Missed Nursing Care Among Emergency and Critical Care Nurses: An Item‐Level Network Analysis With Computer‐Simulated Interventions

**DOI:** 10.1155/jonm/5019343

**Published:** 2026-09-27

**Authors:** Fanfan Lv, Na Wang, Yan Cao, Jia Jia

**Affiliations:** ^1^ Taihe Hospital, Affiliated Hospital of Hubei University of Medicine, Shiyan, China, taihehospital.com; ^2^ School of Nursing, Hubei University of Medicine, Shiyan, China, hbmu.edu.cn

**Keywords:** bridge expected influence, emergency and critical care nursing, missed nursing care, network analysis, NodeIdentifyR, threshold perturbation

## Abstract

**Aim:**

The primary aim was to estimate the item‐level conditional association network of missed nursing care among emergency and critical care nurses. Secondary aims were to identify central, bridge and perturbation‐responsive activities and assess sensitivity to dose, parameterisation and dichotomisation.

**Background:**

Missed nursing care, defined as required care that is omitted, delayed or incomplete, is associated with poorer care quality and adverse patient outcomes. Studies in emergency and critical care have mainly examined total scores, item occurrence or associated factors. How individual omissions are conditionally related and whether frequently missed activities are also central, bridge care categories or respond most strongly to simulated perturbation remain unclear.

**Methods:**

This study was a secondary analysis based on a previously established dataset of 3351 emergency and critical care nurses recruited through stratified cluster sampling from 22 tertiary Grade A hospitals in Hubei Province, China, between January and August 2025. Participants completed Part A of the MISSCARE Survey, comprising 24 missed care activities. Responses were dichotomised as no occurrence or any occurrence. An Ising network estimated conditional associations between binary activities. Expected influence (EI) measured signed overall connectivity, whereas bridge EI (BEI) measured signed connectivity with activities outside each care community. Bootstrap procedures assessed accuracy and stability. Using the NodeIdentifyR algorithm (NIRA), each calibrated node threshold was altered, in turn, in aggravating and alleviating directions, and exact changes in model‐implied mean activation were calculated across all 2^24^ possible states. Dose and alternative parameterisation analyses assessed robustness. A Gaussian graphical model (GGM) estimated sparse partial correlations among the original 0–4 scores to assess sensitivity to dichotomisation.

**Results:**

The Ising network retained 109 of 276 possible edges, of which 108 were positive. Vital sign monitoring had the highest raw EI (8.97), followed by rehabilitation guidance and focused reassessment/care‐plan updating. Pressure ulcer/wound/ostomy care had the highest raw BEI (6.94), followed by rehabilitation guidance and discharge planning/teaching. From a calibrated model‐implied baseline of 4.93 active nodes, a 2‐SD threshold increase for intravenous/drainage tube care yielded the largest aggravating increase in the mean number of active nodes (+5.67), whereas a 2‐SD threshold decrease for emotional support yielded the largest alleviating decrease (−2.70). Emotional support and bathing/skin care were the only activities that remained among the five highest‐ranked alleviating nodes at every examined dose in both parameterisations. BEI rankings were highly concordant between the Ising network and GGM (Spearman’s *ρ* = 0.961), with four of the five highest‐ranked activities shared.

**Conclusions:**

Missed nursing care formed a predominantly positive network. Frequently missed, central, bridge and perturbation‐responsive activities were related but distinct. The NIRA findings are hypotheses derived from a cross‐sectional model and do not represent observed intervention effects or causal estimates.

**Implications for Nursing Management:**

Occurrence frequency alone is insufficient for prioritisation. Centrality, bridge connectivity and simulated responsiveness provide complementary criteria for nursing audits and prospective evaluation. Emotional support and bathing/skin care warrant evaluation in longitudinal studies and intervention trials.

## 1. Introduction

Missed nursing care refers to any required nursing activity that is omitted, delayed or left incomplete [1]. Related terms such as unfinished nursing care and care left undone emphasise required care that remains incomplete, whereas missed nursing care explicitly encompasses complete omission, delayed delivery and incomplete performance [2, 3]. As an error of omission, missed nursing care is widely regarded as a sensitive indicator of nursing care quality. A recent systematic review and meta‐analysis of 28 studies from 14 countries found that the reported prevalence of missed nursing care varied widely across studies, with a median estimate of 56.4% [4]. Missed nursing care has been associated with lower patient satisfaction and increased risks of medication errors, healthcare‐associated infections, falls, pressure injuries, readmissions and mortality [5, 6]. It has also been associated with lower job satisfaction, burnout and intention to leave among nurses [7]. Missed nursing care is therefore an important concern for both patient safety and workforce stability.

These concerns are particularly acute in emergency and critical care settings. Patients in these settings are often unstable and highly dependent on nursing care, whereas nurses must respond to unpredictable patient inflow, rapid changes in clinical condition, frequent interruptions and multiple competing time‐sensitive demands. Assessment, monitoring, treatment support, documentation, basic care and communication must be coordinated within narrow time frames. Delayed observations may impede the timely recognition of deterioration, whereas incomplete documentation or handover may disrupt clinical decision‐making and continuity of care [8]. Recent intensive care studies have reported substantial levels of missed nursing care, particularly in basic care activities, and have linked these omissions to high workload, staffing constraints and changing patient needs [9–11]. The combination of high clinical risk, competing demands and limited tolerance for delay makes emergency and critical care a priority setting for examining not only which activities are missed but also how specific omissions are related.

The missed nursing care model provides a theoretical basis for understanding these relationships [1]. The model identifies labour resources, material resources, communication and teamwork as organisational conditions that influence nurses’ capacity to complete required care. When care demands exceed available resources, nurses’ values, beliefs and work habits affect how activities are prioritised. These decisions occur across the related nursing processes of assessment, planning, intervention and evaluation [12, 13]. Nursing activities therefore do not occur independently. They draw on the same limited time, staffing, equipment and information and are linked through the sequence of care delivery. Assessment and monitoring inform subsequent planning and intervention, whereas documentation and handover support continuity across caregivers. When workload or patient acuity increases, immediately urgent activities may be prioritised, whereas basic, psychosocial, educational or planning activities are deferred [14]. Shared resource constraints, prioritisation decisions and dependencies within the nursing process may consequently produce recurring patterns of co‐occurring omissions.

Most previous research has examined missed nursing care using prevalence estimates, aggregate scores or regression models relating overall missed nursing care to its antecedents and outcomes [7, 9]. These approaches are useful for quantifying the overall burden and identifying associated factors, but they provide limited information about relationships among individual care activities. An aggregate score combines clinically distinct omissions, such as delayed vital sign monitoring, missed emotional support, incomplete basic care and postponed discharge teaching, and treats them as equivalent contributions to a single measure. This may conceal differences in the roles of individual activities and the ways in which they are connected [15, 16]. Regression models based on an aggregate score likewise cannot determine which activities remain associated after the other activities are considered or which activities connect different areas of nursing care. Person‐centred methods such as latent profile analysis can distinguish groups of nurses with different response patterns but do not estimate relationships among the care activities themselves [17]. Consequently, it remains unclear whether particular missed nursing care activities occupy central positions, connect otherwise distinct categories of care or may warrant priority in subsequent intervention research.

Network analysis is suited to these questions because it retains individual missed nursing care activities as distinct components of the system. In a network model, nodes represent individual activities, and edges represent conditional associations between pairs of activities after accounting for the remaining nodes [15, 18]. Centrality indices identify activities that are strongly connected across the network, whereas bridge centrality identifies activities that connect different care categories [19, 20]. Network analysis can therefore reveal aspects of the item‐level organisation of missed nursing care that are not represented by an aggregate score, including closely connected activities and potential links among basic care, individual needs, assessment and planning. Because the network is estimated from cross‐sectional data, however, its edges represent conditional associations rather than causal relationships.

Network structure alone cannot determine how the fitted model would respond to a change in a particular activity. A highly central or bridging activity is not necessarily the activity whose modification would produce the largest projected change in overall network activation [21, 22]. The NodeIdentifyR algorithm (NIRA), introduced by Lunansky et al. [22], addresses this question through model‐based threshold perturbation. The threshold of each node is altered in turn, and the corresponding change in overall network activation is estimated, whereas the remaining model parameters are held constant. This procedure enables candidate activities to be compared according to their model‐implied responsiveness, but it does not reproduce an actual clinical or organisational intervention and cannot establish causal effectiveness. Item‐level network analysis combined with NIRA has rarely been applied to missed nursing care. It therefore remains uncertain whether the most central activities, the activities connecting different care categories and those producing the largest model‐implied responses represent the same or different priorities.

Accordingly, the present study used item‐level MISSCARE Survey data from emergency and critical care nurses to (1) estimate the network structure of missed nursing care activities; (2) identify central and bridge activities within the network; (3) compare candidate targets using the model‐based threshold perturbations implemented in NIRA; and (4) evaluate the sensitivity of the network findings using a Gaussian graphical model (GGM) based on the original ordered responses. The study aimed to clarify the item‐level organisation of missed nursing care and identify priorities for subsequent longitudinal and intervention research in emergency and critical care settings.

## 2. Methods

### 2.1. Participants

This study represents a secondary analysis of data from a multicentre cross‐sectional survey conducted from January to August 2025 among emergency and critical care nurses in Hubei Province, China. Detailed recruitment and data collection procedures for the source survey have been reported previously in a study examining latent profiles and profile‐specific networks of perceived patient safety culture [23]. A stratified cluster sampling strategy was used for the source survey. All 86 tertiary Grade A hospitals in Hubei Province constituted the sampling frame and were stratified according to geographical location within the province. Within each stratum, hospitals were selected using computer‐generated random numbers. Ultimately, 22 hospitals were included. In each selected hospital, all emergency and critical care nurses who met the eligibility criteria were invited to participate.

Eligible nurses were recruited from the emergency department, intensive care unit, cardiac care unit, respiratory intensive care unit, neonatal intensive care unit, paediatric intensive care unit, emergency intensive care unit and neurological intensive care unit. The inclusion criteria were as follows: (a) holding a valid nursing practice certificate; (b) currently providing direct patient care in one of these emergency or critical care departments or units and having at least 1 year of clinical nursing experience; and (c) having received information about the study and voluntarily agreeing to participate. The participant‐level exclusion criteria were as follows: (a) being an intern, trainee or nurse undertaking standardised training and (b) being on sick, maternity or personal leave during the survey period.

### 2.2. Sample Size

The required sample size was initially estimated using the single‐proportion formula for cross‐sectional studies: *n* = $Z_{\alpha /2}^{2}p$ (1 − *p*)/*δ* ^2^ [24]. Because no consistent prior estimate was available for the proportion of emergency and critical care nurses reporting one or more missed nursing care activities in comparable settings, *p* was set at 0.50 to yield the maximum required sample size. With *α* = 0.05, *Z*_*α*/2_ = 1.96 and an absolute precision of *δ* = 0.03, the initial estimate was 1067.1 participants. After applying the prespecified design effect of 1.5 for the multicentre sampling design and allowing for an anticipated 20% invalid‐response rate, the minimum required sample size was 1067.1 × 1.5/(1 − 0.20) = 2000.8, rounded up to 2001. The final analytic sample comprised 3351 nurses, exceeding the prespecified survey‐based minimum of 2001.

For the network analysis, the prespecified regularised pairwise Ising model comprised 24 nodes and 276 possible pairwise interactions. Network‐specific sample‐size requirements depend on the hypothesised network structure and parameter values, the chosen measure of estimation performance and its target value and the probability with which that target should be achieved; they are therefore most appropriately evaluated using a tailored Monte Carlo design [25]. Because no defensible population network specification was available a priori for item‐level missed nursing care in this setting, a model‐specific Monte Carlo sample‐size analysis was not undertaken, as doing so would have required unsupported assumptions about the underlying network structure and parameters. Accordingly, no fixed participant‐to‐parameter ratio was used, and the achieved sample was not presented as establishing a priori network adequacy. The precision and stability of the network estimates in the achieved sample were evaluated empirically using 1000 participant‐level nonparametric bootstrap replicates to assess edge‐weight accuracy and 1000 case‐dropping bootstrap replicates to assess centrality stability. Percentile‐based 95% confidence intervals and correlation stability (CS) coefficients were reported as indicators of estimation uncertainty and stability, respectively [18].

### 2.3. Data Collection

Data were collected using WenJuanXing, a widely used online survey platform in China (https://www.wjx.cn), which generated the questionnaire links and QR codes. Before data collection, the principal investigator provided the nursing directors of the participating hospitals with standardised information on the study purpose, eligibility criteria, survey procedures and ethical requirements. Following administrative permission, designated nursing managers or departmental coordinators identified emergency and critical care nurses who met the eligibility criteria and distributed the questionnaire through departmental WeChat groups. All nurses identified as eligible were invited to participate. Participation was voluntary, and responses were collected anonymously. The numbers screened, found eligible and invited were not recorded centrally. All questionnaire items were mandatory, and submission was permitted only after all items had been completed. Only one submission was permitted for each combination of account and IP address. After data collection, questionnaires were excluded if the complete response record was identical to that of another submission, an invariant response pattern was observed across the full set of multi‐item scales or the reported years of nursing experience exceeded the maximum possible based on the participant’s age. A total of 3392 questionnaires were submitted. Of these, 41 met at least one questionnaire‐level exclusion criterion, leaving 3351 questionnaires for the final analysis, representing 98.8% of all submitted questionnaires.

### 2.4. Ethical Considerations

This study was conducted in accordance with the Declaration of Helsinki and all relevant guidelines and regulations. Ethical approval was obtained from the Medical Ethics Committee of Shiyan Taihe Affiliated Hospital of Hubei University of Medicine (Ethics Approval No. 2024KS53). Before participation, all participants were informed of the study purpose, procedures, voluntary nature of participation and confidentiality measures. Electronic informed consent was obtained from each participant before access to the questionnaire. Participants provided consent by selecting the ‘Agree to participate in this survey’ option. The survey was administered anonymously, and all responses were submitted directly to the research team through the online platform to maintain confidentiality.

### 2.5. Measurements

#### 2.5.1. Demographic and Work‐Related Information

A self‐designed questionnaire was used to collect participants’ demographic and work‐related characteristics. Demographic variables included gender, age, marital status, educational level and professional title. Work‐related variables included years of nursing experience, weekly working hours, self‐rated sleep quality during the preceding week (poor, fair or good) and the frequency of workplace violence experienced during the preceding year (0, 1, 2–3 or ≥ 4 times).

#### 2.5.2. Missed Nursing Care

Missed nursing care was assessed using Part A of the MISSCARE Survey, developed by Kalisch and Williams [26] to assess the frequency with which nursing care activities are omitted, delayed or left incomplete in hospital settings. The scale was translated and culturally adapted for use among Chinese hospital nurses by Si [27]. The Chinese version comprises 24 items rated on a five‐point Likert scale from 0 (‘never missed’) to 4 (‘always missed’). The scale is scored as a single total score, obtained by summing the 24‐item scores, with a possible range of 0–96. Higher scores indicate more frequent missed nursing care, whereas a total score of 0 indicates that no missed care was reported across the 24 activities. In the Chinese validation study, the scale‐level content validity index was 0.98, the item‐level content validity indices ranged from 0.83 to 1.00, the test–retest reliability coefficient was 0.914, and Cronbach’s *α* was 0.924. In the present sample, Cronbach’s *α* for the original 0–4 item responses was 0.970, and the corrected item–total correlations ranged from 0.716 to 0.903.

Following the care‐activity classification used in the MISSCARE Survey and related MISSCARE studies, the 24 items were assigned to four content‐based communities for network analysis: basic care, individual needs, assessment and planning. These communities were used to define node groups for network interpretation, visualisation and bridge centrality analysis and should not be interpreted as validated subscales or dimensions of the Chinese version. In the item labels, PRN denotes medication administered as needed, and IV denotes intravenous. Basic care included bed linen care, turning every 2 h, mouth care, bathing/skin care, pressure ulcer/wound/ostomy care and hand hygiene. Individual needs included toileting assistance, emotional support, PRN medication, timely medication administration and call light response. Assessment included focused reassessment/care‐plan updating, IV/drainage tube care, risk assessment, intake/output monitoring, vital sign monitoring, bedside glucose monitoring, nursing documentation, medical record review and ward rounds. Planning included dietary guidance, health education, rehabilitation guidance and discharge planning/teaching. The full item wording, short labels used in the figures and content‐based communities are provided in Supporting Table S1.

### 2.6. Data Analysis

#### 2.6.1. Descriptive Analysis

Participant characteristics were summarised using frequencies and percentages. For each missed nursing care item, the number and percentage of responses in each of the five ordered categories were calculated. Response categories ranged from 0 (‘never missed’) to 4 (‘always missed’), with higher values indicating more frequent missed nursing care. There were no missing responses for any of the 24 items. For the item‐level occurrence analysis, a response of 0 was coded as 0, indicating no reported occurrence, whereas responses of 1–4 were coded as 1, indicating any reported occurrence. The occurrence proportion for each item was calculated as the percentage of participants with a dichotomised value of 1. All analyses were conducted using R version 4.5.2 (R Foundation for Statistical Computing, Vienna, Austria). The principal packages were IsingFit version 0.4, qgraph version 1.9.8, bootnet version 1.8, networktools version 1.6.0, IsingSampler version 0.2.4, mgm version 1.2–15 and nodeIdentifyR version 1.0.0. Exact state enumeration, model calibration and threshold‐perturbation calculations for the NIRA were implemented using custom R code.

#### 2.6.2. Ising Network Estimation

Consistent with the study objective of characterising the overall item‐level structure of missed nursing care in the multicentre sample, the primary analysis targeted a single pooled network of the 24 items across all nurses from the 22 participating hospitals. The hospital structure was not modelled as a separate level. The pooled network was estimated from the dichotomised item responses. A response of 0 (‘never missed’) represented no reported occurrence, whereas responses of 1–4 represented any reported occurrence. This cut‐off corresponded to the substantive distinction between the absence and occurrence of each missed nursing care activity, applied a consistent definition across all items and was consistent with occurrence‐based scoring in missed nursing care research [2, 28]. The resulting binary variables also satisfied the two‐state data requirements of the Ising model and the NIRA implementation used in this study. Accordingly, the Ising and NIRA analyses examined no reported occurrence versus any reported occurrence of each care activity. The original 0 to 4 responses were retained for the descriptive analysis and GGM sensitivity analysis.

A pairwise Ising model was fitted in which the joint distribution was parameterised by a threshold for each node and an interaction parameter for each pair of nodes. For any binary response pattern, its unnormalised log probability was defined by the threshold contributions of the active nodes and the interaction contributions of active node pairs. Under this parameterisation, higher thresholds indicated a greater propensity for node activation, positive interaction parameters indicated positive conditional associations, and negative interaction parameters indicated inverse conditional associations after adjustment for all remaining nodes. The network was estimated using eLasso in IsingFit, which combines nodewise l1‐regularised logistic regression with extended Bayesian information criterion model selection [29]. The EBIC tuning parameter was set to 0.25, and the AND rule was applied so that an edge was retained only when the corresponding association was selected in both nodewise regressions. The network was visualised using qgraph with a spring layout [30]. Edge‐weight accuracy was assessed using 1000 participant‐level nonparametric bootstrap samples, from which percentile‐based 95% bootstrap intervals were obtained [18].

#### 2.6.3. Centrality and Bridge Centrality

Strength and one‐step expected influence (EI) were calculated as standard centrality indices. Strength was defined as the sum of the absolute weights of all edges connected to a node, whereas EI was defined as the algebraic sum of those edge weights and therefore retained their signs [20]. Bridge EI (BEI) was calculated using four prespecified content‐based communities: basic care, individual needs, assessment and planning. It was defined as the sum of the edge weights connecting a node to nodes outside its own community [19]. Raw, unstandardised EI and BEI values were used for numerical reporting and ranking.

The stability of strength and EI was assessed using case‐dropping bootstrap analysis with 1000 iterations. The CS coefficient represented the maximum proportion of participants that could be removed while retaining, with 95% probability, a correlation of at least 0.70 between the subset and full‐sample estimates. Coefficients should be at least 0.25 and preferably above 0.50 [18]. BEI was additionally assessed using 1000 nonparametric bootstrap samples and 1000 case‐dropping bootstrap samples, with the eLasso network re‐estimated using the same settings in each sample. The nonparametric bootstrap distributions were used to obtain percentile‐based 95% bootstrap intervals and to calculate the frequency with which each node ranked among the five highest BEI values. For the case‐dropping analysis, 1000 samples were generated, comprising 100 samples at each of 10 drop proportions ranging from 5% to 75%. The BEI CS coefficient was calculated using the same criterion of a correlation of at least 0.70 in 95% of the samples. Individual participants, rather than hospitals, were used as the resampling units in all bootstrap analyses.

#### 2.6.4. NodeIdentifyR Threshold‐Perturbation Analysis

The NIRA was implemented according to the original model‐based threshold‐perturbation framework described by Lunansky et al. [22], with moderation‐effect testing, permutation testing and repeated‐simulation stability assessment following Wang et al. [31]. The original eLasso model estimated from the full sample, as described in Section 2.6.2, served as the primary NIRA model after common‐offset calibration. Under the joint Ising parameterisation used for the exact calculations, the uncalibrated eLasso model did not reproduce the observed mean number of active nodes. Because the primary NIRA outcome was the change in the expected total number of active nodes, the observed mean of this quantity was selected as the calibration target. Before perturbation, the eLasso interaction parameters were held fixed and a single common offset was added to all node thresholds. Using a common rather than node‐specific offset provided a parsimonious global adjustment that preserved all interaction parameters, the between‐node differences in thresholds and the standard deviation across the threshold values used to scale the perturbation doses. It therefore adjusted the overall activation propensity without re‐estimating the relative threshold structure of the original eLasso model.

The common offset was determined through numerical root finding based on exact enumeration of all 2^24^ (16,777,216) possible binary states, such that the exact model‐implied expected number of active nodes equalled the observed mean number of dichotomised items coded as 1. This calibration targeted only agreement in overall mean activation and was not intended to force agreement in individual node activation probabilities or in the complete binary sum‐score distribution. Residual model fit after calibration was therefore evaluated by comparing the observed and model‐implied node activation probabilities using the Pearson correlation across the 24 nodes, mean absolute error and maximum absolute error; the observed and model‐implied means and standard deviations of the number of active nodes; the probabilities of binary sum scores of 0 and 24; and the total variation and Kolmogorov–Smirnov distances between the observed and model‐implied binary sum‐score distributions. The model‐implied mean sum score was defined as the expected number of active nodes, and the model‐implied mean activation proportion was calculated by dividing this value by 24.

To assess the fixed‐interaction assumption underlying the threshold‐perturbation analysis, each of the 24 nodes was examined in turn as a potential moderator of pairwise associations using the moderated network procedure implemented in mgm. The EBIC tuning parameter was set to 0.25, the AND rule was applied, and 1000 participant‐level nonparametric bootstrap samples were generated for each candidate moderator. A moderation effect was flagged when its percentile‐based 95% bootstrap interval excluded zero.

In the primary perturbation analysis, one node threshold was altered at a time, whereas the other 23 thresholds and all interaction parameters were held constant. An increase equal to twice the standard deviation across the 24 calibrated node thresholds represented an aggravating perturbation, whereas a decrease in the same magnitude represented an alleviating perturbation. The model‐implied mean sum score and mean activation proportion for the baseline and each perturbed condition were calculated exactly over the complete state space. Nodes were ranked separately within each perturbation direction according to the absolute magnitude of change from the calibrated baseline. The resulting estimates and rankings were therefore based on exact model expectations rather than Monte Carlo averages.

Permutation validation of the primary 2‐SD analysis assessed whether differences in model‐generated mean sum scores between the calibrated baseline and each perturbed condition exceeded the variation expected under random reassignment of condition labels. For the baseline and each perturbed condition, 5000 total scores were generated from the corresponding exact model‐implied sum‐score distribution, and 5000 two‐sided label permutations were performed for each node. Permutation *p* values were calculated using a plus‐one correction and adjusted using the Bonferroni method across the 24 node‐level tests separately within each perturbation direction. Repeated‐simulation stability was assessed across 1000 repetitions, with 5000 model‐generated total scores per condition in each repetition. Absolute mean‐score changes were ranked separately within each perturbation direction, and the frequencies with which each node ranked first or among the five highest‐ranked nodes were calculated. These procedures assessed permutation‐based differences and ranking stability conditional on the fitted model‐implied distributions; they were not additional tests of empirical model fit and could not correct residual distributional misfit.

Parameterisation sensitivity was assessed using a postselection model in which the edge structure selected by eLasso was retained and the corresponding parameters were re‐estimated without penalisation. The postselection model was separately calibrated to the observed mean number of active nodes using the same exact common‐offset procedure. At 2 SD, agreement between the calibrated original eLasso and postselection models was evaluated using Spearman correlations between their effect‐magnitude rankings and overlap between their sets of five highest‐ranked nodes within each perturbation direction. Dose sensitivity was assessed in both calibrated models by repeating the exact analysis using threshold changes of 0.5, 1.0, 1.5 and 2.0 times the model‐specific standard deviation across the node thresholds in both perturbation directions. Cross‐dose consistency was assessed by identifying nodes that remained among the five highest‐ranked nodes at all four perturbation doses in both models. All NIRA quantities were conditional on the fitted cross‐sectional Ising model. They were not treated as observed item prevalence estimates, changes in the original MISSCARE total score, causal intervention effects or evidence of real‐world intervention efficacy.

#### 2.6.5. GGM Sensitivity Analysis

Using the same pooled multicentre sample, a GGM was estimated from the original 0 to 4 item responses to assess the sensitivity of the network findings to dichotomisation. A Spearman correlation matrix was used because the item responses were ordered and nonnormally distributed. A sparse partial‐correlation network was estimated using EBICglasso with the EBIC tuning parameter set to 0.50 [18, 32]. The same four content‐based communities were used to calculate BEI. Edge‐weight accuracy was assessed using 1000 participant‐level nonparametric bootstrap samples, and the stability of strength and EI was assessed using 1000 case‐dropping bootstrap iterations. Agreement between the Ising and GGM results was evaluated using overlap in retained nonzero edges, the Jaccard index, Spearman correlations between the corresponding strength, EI and BEI rankings and overlap among the five highest‐ranked nodes for EI and BEI.

## 3. Results

### 3.1. Descriptive Statistics

A total of 3351 emergency and critical care nurses were included in this study. Most participants were female (*n* = 3,139, 93.7%), and the largest age group was 26–35 years (*n* = 1,663, 49.6%). Most nurses were married (*n* = 2,612, 77.9%), held an intermediate professional title (*n* = 1,852, 55.3%) and had a bachelor’s degree (*n* = 2,731, 81.5%). Regarding occupational characteristics, the largest subgroup had 11–15 years of work experience (*n* = 915, 27.3%). Nearly half reported fair sleep quality during the preceding week (*n* = 1,649, 49.2%), and 1417 (42.3%) reported experiencing workplace violence two to three times during the preceding year. Most participants worked 41–50 h per week (*n* = 2,292, 68.4%). Detailed demographic and occupational characteristics are presented in Table 1.

**TABLE 1:** The characteristics of the participants (*N* = 3351).

Variables	Classification	*N* (%)
Gender	Male	212 (6.3)
	Female	3139 (93.7)

Age (years)	≤ 25	330 (9.8)
	26–35	1663 (49.6)
	36–45	997 (29.8)
	≥ 46	361 (10.8)

Work experience (years)	≤ 5	655 (19.5)
	6–10	875 (26.1)
	11–15	915 (27.3)
	16–20	570 (17.0)
	≥ 21	336 (10.0)

Marital status	Unmarried	695 (20.7)
	Married	2612 (77.9)
	Divorced or widowed	44 (1.3)

Professional title	Junior level	951 (28.4)
	Intermediate level	1852 (55.3)
	Associate senior title or above	548 (16.3)

Education	Junior college degree and below	385 (11.5)
	Bachelor’s degree	2731 (81.5)
	Master’s degree and above	235 (7.0)

Sleep quality during the past week	Poor	1191 (35.5)
	Fair	1649 (49.2)
	Good	511 (15.2)

Frequency of workplace violence	0	564 (16.8)
	1	1155 (34.5)
	2–3	1417 (42.3)
	≧ 4	215 (6.4)

Weekly working hours	≤ 40 h	572 (17.1)
	41–50 h	2292 (68.4)
	≥ 51 h	487 (14.5)

**FIGURE 1:**
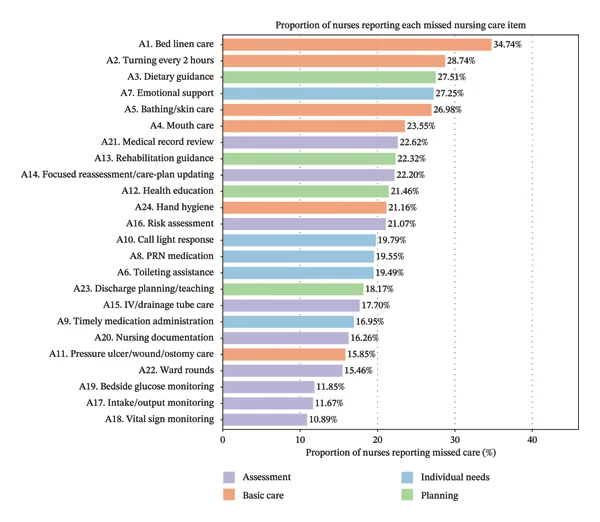
Item‐level occurrence proportions for the 24 missed nursing care activities. Note. Each bar represents the proportion of nurses who reported that the corresponding nursing care activity had been missed at least once. Original responses were dichotomised as 0 (‘never missed’) = not missed and 1–4 (‘rarely missed’ to ‘always missed’) = missed. Bar colours indicate the four content‐based communities used in the network analysis: basic care, individual needs, assessment and planning.

The full item wording corresponding to each node, the short labels used in the figures and the content‐based community assignments are provided in Supporting Table S1. Supporting Table S2 presents the original 0–4 response scale and category definitions, scoring direction, exact dichotomisation rule and frequencies and percentages for each response category across all 24 missed nursing care items. All 3351 participants provided complete responses to all 24 items. For the dichotomised analysis, an original response of 0 (‘never missed’) was coded as 0, whereas responses of 1–4 (‘rarely missed’ to ‘always missed’) were coded as 1, indicating that the activity had been missed at least once. Item‐level occurrence proportions ranged from 10.89% to 34.74%, with an unweighted arithmetic mean of 20.55% across the 24 items (Figure 1). The highest occurrence proportions were observed for bed linen care (A1, 34.74%), turning every 2 h (A2, 28.74%), dietary guidance (A3, 27.51%), emotional support (A7, 27.25%) and bathing/skin care (A5, 26.98%). The lowest occurrence proportions were observed for vital sign monitoring (A18, 10.89%), intake/output monitoring (A17, 11.67%) and bedside glucose monitoring (A19, 11.85%).

### 3.2. Network Structure and Bridge Centrality of Missed Nursing Care

**FIGURE 2:**
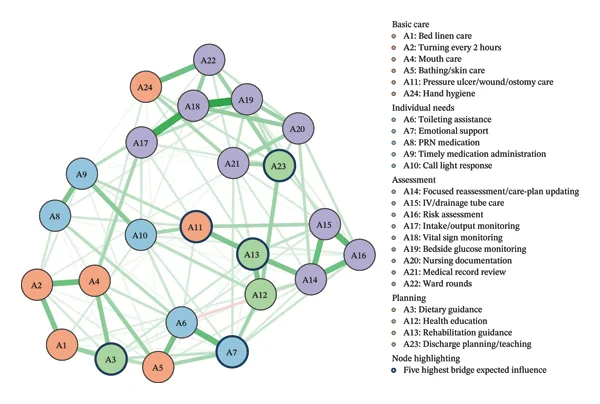
Ising network of missed nursing care items. Note. Nodes represent individual missed nursing care activities, and edges represent pairwise conditional associations after accounting for all remaining nodes in the network. Edge thickness reflects the magnitude of each conditional association. Green edges indicate positive conditional associations, whereas red edges indicate negative conditional associations. Node fill colours indicate the four care‐activity communities: basic care, individual needs, assessment and planning. Dark‐blue outlines identify the five nodes with the highest bridge expected influence.

The Ising network of the 24 missed nursing care items is presented in Figure 2. Nodes were coloured according to the four care‐activity communities, and dark‐blue outlines indicate the five nodes with the highest BEI. Of the 276 possible pairwise associations, 109 were retained as nonzero edges, of which 108 were positive and one was negative. The largest edge weights were observed between vital sign monitoring and bedside glucose monitoring (A18–A19, weight = 2.76), intake/output monitoring and vital sign monitoring (A17–A18, weight = 2.52), IV/drainage tube care and risk assessment (A15–A16, weight = 2.12), toileting assistance and emotional support (A6–A7, weight = 2.01) and focused reassessment/care‐plan updating and IV/drainage tube care (A14–A15, weight = 1.98).

**FIGURE 3:**
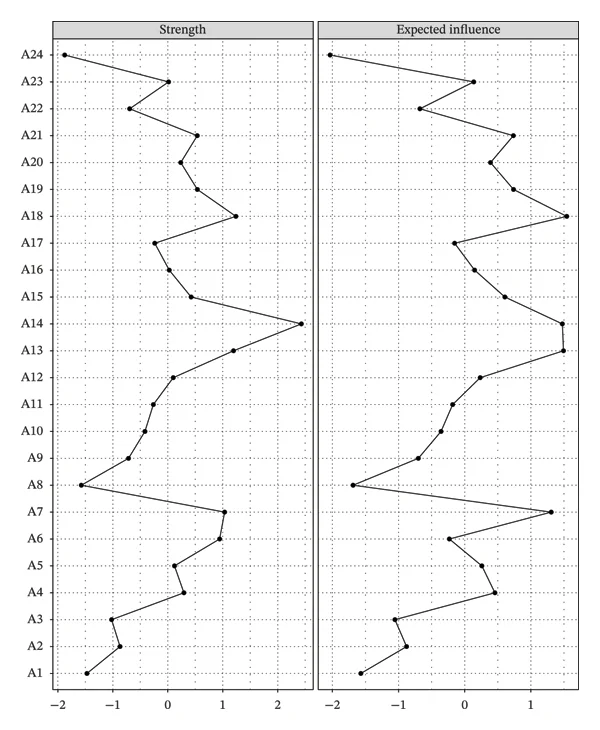
Centrality indices of missed nursing care items in the Ising network. Note. Strength represents the sum of the absolute weights of all edges connected to each node, whereas expected influence represents the algebraic sum of those edge weights and therefore retains their signs. Values are presented as standardised z‐scores, with higher values indicating greater relative importance within the network.

Centrality estimates are shown in Figure 3. The five nodes with the highest raw EI were vital sign monitoring (A18, 8.97), rehabilitation guidance (A13, 8.92), focused reassessment/care‐plan updating (A14, 8.90), emotional support (A7, 8.73) and bedside glucose monitoring (A19, 8.13). The five nodes with the highest strength were focused reassessment/care‐plan updating (A14), vital sign monitoring (A18), rehabilitation guidance (A13), emotional support (A7) and toileting assistance (A6). Bootstrap assessments of edge‐weight accuracy and centrality stability are presented in Supporting Figures S1 and S2, respectively. The participant‐level case‐dropping bootstrap yielded CS coefficients of 0.594 for EI and 0.439 for strength.

**FIGURE 4:**
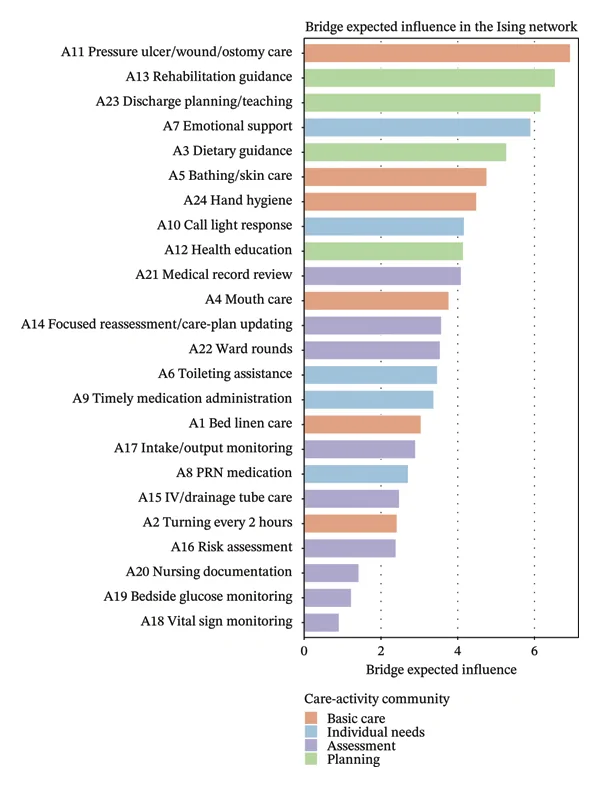
Bridge expected influence of missed nursing care items in the Ising network. Note. All 24 nodes are displayed in descending order of bridge expected influence, and bar colours indicate the four care‐activity communities. Bridge expected influence was calculated as the algebraic sum of the edge weights connecting a node to nodes outside its own community. Values are presented as raw, unstandardised bridge expected influence values. Higher values indicate greater net positive connectivity with nodes in other care‐activity communities.

The BEI ranking differed from the standard centrality rankings. The five nodes with the highest BEI were pressure ulcer/wound/ostomy care (A11, BEI = 6.94), rehabilitation guidance (A13, 6.54), discharge planning/teaching (A23, 6.17), emotional support (A7, 5.90) and dietary guidance (A3, 5.28). These nodes are highlighted by dark‐blue outlines in Figure 2, and the complete ranking of all 24 nodes is presented in Figure 4. In the nonparametric bootstrap analysis, the frequencies with which A11, A13, A23, A7 and A3 ranked among the five highest BEI values were 99.8%, 95.8%, 89.8%, 82.8% and 67.8%, respectively. The case‐dropping bootstrap yielded a CS coefficient of 0.750 for BEI, indicating good stability. EI and BEI shared two nodes among their respective five highest‐ranked nodes: rehabilitation guidance (A13) and emotional support (A7). The other three leading bridge nodes comprised one basic care activity—pressure ulcer/wound/ostomy care (A11)—and two planning activities—discharge planning/teaching (A23) and dietary guidance (A3); no assessment activity ranked among the five highest BEI values.

### 3.3. NIRA Threshold‐Perturbation Analysis

The observed mean number of active nodes was 4.931961, corresponding to an unweighted mean activation proportion of 20.549836% across the 24 items. Before common‐offset calibration, the original eLasso model yielded an exact model‐implied baseline mean sum score of 1.129673 and a corresponding mean activation proportion of 4.706971%. A positive common offset, *c* = 0.273340, was added to each of the 24 node thresholds. Following calibration, the exact model‐implied baseline mean sum score was 4.931961, with a mean activation proportion of 20.549836%, matching the observed mean to six decimal places. Across the 24 nodes, the Pearson correlation between the observed and model‐implied activation probabilities was 0.969, with a mean absolute error of 2.63 percentage points and a maximum absolute error of 7.82 percentage points. The observed and model‐implied standard deviations of the total number of active nodes were 7.646 and 6.862, respectively. For binary sum scores of 0 and 24, the observed probabilities were 48.52% and 7.49%, respectively, compared with model‐implied probabilities of 25.22% and 2.16%. The total variation distance was 0.304, and the Kolmogorov–Smirnov distance was 0.233 (Supporting Table S3 and Supporting Figures S3 and S4).

**FIGURE 5:**
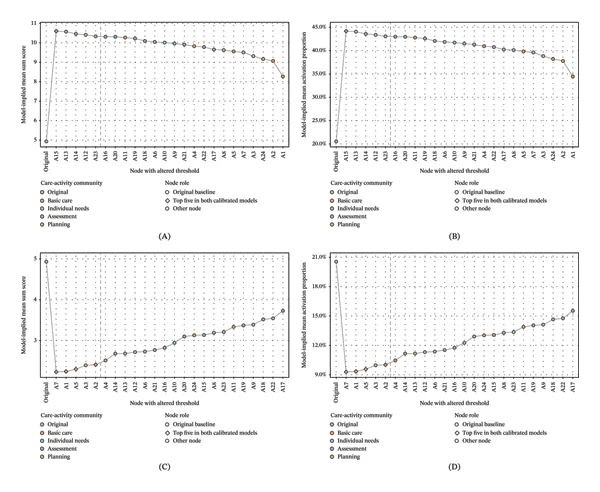
Exact model‐implied effects of node‐specific threshold perturbations in the calibrated original eLasso Ising model. Note. Panels (A and C) show the model‐implied mean sum scores following aggravating and alleviating perturbations, respectively. Panels (B and D) express the corresponding expected values as mean activation proportions by dividing the mean sum scores by 24; they therefore do not represent separate prevalence outcomes. For each condition, the threshold of one target node was altered by 2 SD, whereas all edge weights and the remaining node thresholds were held constant. Dashed vertical lines separate the five largest effects in the primary eLasso model. Diamonds identify targets that were also ranked among the five largest effects in the calibrated postselection sensitivity model (aggravating: A15; alleviating: A7, A1, A5 and A2) and therefore indicate cross‐model ranking agreement rather than statistical significance. Node colours indicate the four content‐based communities. All estimates are model‐implied and should not be interpreted as causal effects or evidence of real‐world intervention efficacy. (A) Model‐implied effects of aggravating threshold perturbations. (B) Increases in model‐implied mean activation proportion. (C) Model‐implied effects of alleviating threshold perturbations. (D) Decreases in model‐implied mean activation proportion.

No moderation‐effect estimate had a percentile‐based 95% bootstrap interval that excluded zero (Supporting Table S4). The exact 2‐SD threshold‐perturbation results for the calibrated original eLasso model are presented in Figure 5 and Supporting Table S5. In the aggravating direction, increasing the threshold of each of the 24 nodes raised the model‐implied mean sum score (Figure 5A, B). The five largest model‐implied increases followed perturbation of IV/drainage tube care (A15: postperturbation mean sum score = 10.60, change = +5.67), rehabilitation guidance (A13: 10.57, +5.64), focused reassessment/care‐plan updating (A14: 10.46, +5.53), health education (A12: 10.41, +5.48) and discharge planning/teaching (A23: 10.33, +5.40). The corresponding postperturbation mean activation proportions were 44.16%, 44.06%, 43.57%, 43.37% and 43.05%, representing increases of 23.61, 23.51, 23.02, 22.82 and 22.50 percentage points from the calibrated baseline, respectively.

In the alleviating direction, decreasing the threshold of each of the 24 nodes reduced the model‐implied mean sum score (Figure 5C, D). The five largest model‐implied decreases followed perturbation of emotional support (A7: postperturbation mean sum score = 2.23, change = −2.70), bed linen care (A1: 2.24, −2.69), bathing/skin care (A5: 2.30, −2.63), dietary guidance (A3: 2.39, −2.54) and turning every 2 h (A2: 2.41, −2.52). The corresponding postperturbation mean activation proportions were 9.29%, 9.34%, 9.57%, 9.97% and 10.03%, representing decreases of 11.26, 11.21, 10.98, 10.58 and 10.52 percentage points from the calibrated baseline, respectively.

In the permutation validation, comparisons of model‐generated mean sum scores between the calibrated baseline and each perturbed condition yielded the minimum attainable Bonferroni‐adjusted *p* value of 0.0048 for all 24 nodes in both directions (Supporting Table S6 and Supporting Figure S5). Across 1000 repeated simulations, A15 and A13 were included among the five highest‐ranked nodes in 97.3% and 96.7% of the aggravating simulations, respectively; A7, A1 and A5 were included among the five highest‐ranked nodes in 100.0%, 100.0% and 99.9% of the alleviating simulations, respectively (Supporting Table S7 and Supporting Figure S6).

At 2 SD, the Spearman correlations between the eLasso and postselection effect‐magnitude rankings were 0.781 in the aggravating direction and 0.851 in the alleviating direction. Only A15 was included among the five highest‐ranked aggravating nodes in both models, whereas A7, A1, A5 and A2 were included among the five highest‐ranked alleviating nodes in both models. Across perturbation magnitudes of 0.5, 1.0, 1.5 and 2.0 SD, only A5 and A7 remained among the five highest‐ranked alleviating nodes in both models (Supporting Tables S8 and S9 and Supporting Figure S7).

### 3.4. GGM Sensitivity Analysis

A GGM was estimated using the original 0–4 item scores to examine whether the main network findings were maintained under an alternative model specification. The GGM retained 161 of the 276 possible edges as nonzero. The CS coefficients were 0.750 for both EI and strength, indicating good stability of the centrality estimates. The estimated GGM, edge‐weight accuracy, case‐dropping stability, centrality indices and BEI estimates are presented in Supporting Figures S8–S12.

The two models showed substantial overlap in their edge structures. Of the 109 nonzero edges retained in the Ising network, 107 were also retained in the GGM. Two edges were unique to the Ising network, whereas the GGM additionally retained 54 edges, resulting in a Jaccard index of 0.656. Spearman’s rank correlations between the corresponding Ising and GGM centrality rankings were 0.814 for strength and 0.626 for EI. Vital sign monitoring (A18) ranked first in EI in both models.

BEI showed a Spearman rank correlation of 0.961 between the two models. Pressure ulcer/wound/ostomy care (A11) ranked first in BEI in both models. Rehabilitation guidance (A13), discharge planning/teaching (A23) and dietary guidance (A3) were also among the five highest‐ranked bridge nodes in both models. Thus, four of the five highest‐ranked bridge nodes, including the highest‐ranked node, were shared across the two models. Emotional support (A7) was included among the five highest‐ranked bridge nodes in the Ising network, whereas hand hygiene (A24) was included in the GGM. The main bridge‐node pattern was therefore maintained when the original 0–4 item scores were analysed using the GGM.

## 4. Discussion

Using item‐level data from 3351 emergency and critical care nurses, this study examined the network structure of missed nursing care, identified central and bridge activities and compared node‐specific responses to model‐based threshold perturbations. Three findings are noteworthy. First, missed care activities formed a densely connected and predominantly positive network, indicating that omissions tended to occur together rather than as isolated events. Second, central and bridge activities represented different structural roles. Assessment and monitoring activities were prominent in standard centrality, whereas the leading bridge nodes included activities from basic care, planning and individual needs. The bridge findings showed high agreement between the Ising network and the GGM based on the original ordered responses. Third, the NIRA rankings differed by perturbation direction. In the primary eLasso model, assessment and planning activities were prominent under aggravating perturbations, whereas emotional support and several basic care activities were prominent under alleviating perturbations. Agreement across doses and model parameterisations was stronger in the alleviating direction, particularly for bathing/skin care and emotional support. These findings indicate that missed nursing care is not only an overall level of omitted care but also a structured pattern of interrelated activities whose central, bridging and perturbation‐responsive roles are not interchangeable.

### 4.1. Interconnections Among Missed Nursing Care Activities

Of the 109 edges retained in the Ising network, 108 were positive, supporting the view that missed nursing care activities are embedded in a shared work system rather than occurring independently. Most of the strongest edges linked assessment and monitoring activities, including vital sign monitoring, bedside glucose monitoring, intake/output monitoring, focused reassessment/care‐plan updating and IV/drainage tube care. These activities jointly contribute to nursing surveillance, treatment evaluation and recognition of clinical deterioration. Their close conditional associations may therefore reflect the shared time, attention, information and documentation processes on which they depend. The prominence of vital sign monitoring is particularly informative. It had the highest EI despite having the lowest observed occurrence proportion. Frequency describes how often an activity was missed, whereas EI reflects how strongly its omission was conditionally connected with other omissions [21]. Thus, an infrequently missed activity may still occupy an important structural position. The cross‐sectional network does not establish that omission of one monitoring activity causes another to be missed, but it shows that these activities formed a closely connected part of the observed missed‐care pattern.

The occupational characteristics of the sample provide a plausible context for this interconnected pattern. Overall, 82.9% of participants worked more than 40 h per week, and 83.2% reported at least one episode of workplace violence during the preceding year. Previous evidence has linked work schedules characterised by long working hours and insufficient rest, higher nursing workload and less favourable patient‐to‐nurse ratios with more missed nursing care, although associations have varied across measures and settings [14, 33, 34]. Safdari et al. [35], for example, found a positive but statistically nonsignificant association between workload and missed nursing care among intensive care nurses. Under acute time pressure, urgent, measurable and treatment‐related activities may receive priority, whereas recurrent or interaction‐intensive activities such as hygiene, skin care, dietary guidance and emotional support may be deferred [13, 36]. Workplace violence may further disrupt care by limiting therapeutic communication and encouraging shorter, task‐oriented encounters; qualitative evidence has described postponed care, withdrawal from care and reduced nurse–patient interaction following verbal violence [37]. These mechanisms are consistent with the present pattern but were not tested directly, because working hours and workplace violence were not included as external variables in the network model. Future studies could use moderated network models to test whether specific item‐to‐item associations vary with workload or violence exposure and multigroup networks to compare topology and centrality across exposure groups. Longitudinal and multilevel network designs would further help determine whether changes in these organisational conditions precede changes in missed‐care relationships and whether the patterns differ across hospitals.

### 4.2. Distinct Roles of Central and Bridge Activities

The standard centrality and bridge centrality results identified different aspects of the network. Vital sign monitoring, rehabilitation guidance, focused reassessment/care‐plan updating, emotional support and bedside glucose monitoring had the highest EI, indicating strong net positive connectivity with the network as a whole. In contrast, pressure ulcer/wound/ostomy care, rehabilitation guidance, discharge planning/teaching, emotional support and dietary guidance had the highest BEI. Only rehabilitation guidance and emotional support appeared among the five highest‐ranked nodes for both indices. BEI therefore provided information that could not be inferred from standard centrality alone. Pressure ulcer/wound/ostomy care connected basic care with activities outside that community, whereas rehabilitation guidance, discharge planning/teaching and dietary guidance linked planning with other areas of nursing work. Emotional support connected individual needs with the broader missed‐care pattern. These bridge positions indicate cross‐community connectedness; they should not be interpreted as evidence that omissions spread causally from one activity or community to another.

The bridge findings were also the most consistent across model specifications. BEI had a Spearman correlation of 0.961 between the Ising and GGMs. Pressure ulcer/wound/ostomy care ranked first in both, and rehabilitation guidance, discharge planning/teaching and dietary guidance also remained among the five highest‐ranked bridge nodes. In comparison, agreement for standard EI was lower, suggesting that its ranking was more affected by the distinction between binary occurrence and the original ordered frequency scores. The GGM findings reduced, but did not eliminate, concern that dichotomisation determined the bridge pattern. Moreover, consistency between the two models in this sample does not establish that the same topology or node rankings will be observed elsewhere. Comparisons with previous studies should account for differences in measurement instruments, item wording, scoring and cut‐off points, clinical settings, countries, hospital units, sample composition and sampling methods. Differences in item prevalence or network structure across studies may reflect these methodological and contextual features rather than substantive inconsistency.

### 4.3. Direction‐Specific NIRA Findings

The NIRA findings demonstrated clear directional asymmetry. In the calibrated original eLasso model, the five largest model‐implied increases under the 2‐SD aggravating perturbations occurred when the thresholds for IV/drainage tube care, rehabilitation guidance, focused reassessment/care‐plan updating, health education and discharge planning/teaching were increased. All five activities belonged to the assessment or planning community. Conversely, the five largest model‐implied decreases under the corresponding alleviating perturbations occurred when the thresholds for emotional support, bed linen care, bathing/skin care, dietary guidance and turning every 2 h were decreased; three were basic‐care activities, and emotional support was the leading node. The model‐implied aggravating and alleviating responses were therefore not mirror images within the fitted network. The activities showing the greatest perturbation responsiveness were also not identical to those ranked highest on strength, EI or BEI, indicating that structural connectivity alone did not determine the magnitude of the model‐implied response to a specified threshold change. The sensitivity analyses indicated greater robustness of the alleviating rankings. At 2 SD, only IV/drainage tube care was among the five highest‐ranked aggravating nodes in both model parameterisations, and no aggravating node remained among the five highest‐ranked nodes across all examined doses and both models. By comparison, emotional support, bed linen care, bathing/skin care and turning every 2 h were among the five highest‐ranked alleviating nodes in both models at 2 SD, whereas bathing/skin care and emotional support remained among the five highest‐ranked nodes at every dose in both parameterisations.

The common‐offset recalibration aligned the model‐implied baseline mean activation with the observed mean while preserving the relative threshold differences and interaction parameters used in the perturbation analysis. It did not, however, reproduce the complete observed total‐score distribution. The model‐implied distribution was less dispersed than the observed distribution and assigned lower probabilities to both the all‐zero and all‐active response patterns. Because the NIRA effects were calculated over the fitted distribution of binary states, the residual differences in the allocation of probability across total scores may have affected the absolute magnitudes of the estimated changes and potentially the relative node rankings. Exact state enumeration eliminated Monte Carlo error from the primary estimates, whereas permutation testing and repeated simulations assessed conditional differences and ranking stability; none of these procedures addressed the uncertainty arising from residual model misfit. The greater cross‐model and cross‐dose consistency of bathing/skin care and emotional support therefore provide comparatively stronger support for considering them as candidates for prospective evaluation, whereas the more model‐sensitive aggravating rankings should be interpreted with greater caution. These findings remain conditional, hypothesis‐generating projections from a cross‐sectional Ising model and do not demonstrate changes in the original MISSCARE score, causal effects or real‐world intervention efficacy. Longitudinal observational studies and, subsequently, prospective intervention studies are needed to determine whether interventions designed to reduce omission of the identified care activities alter subsequent patterns of missed nursing care.

### 4.4. Implications for Nursing Management and Research

The findings support item‐level monitoring alongside conventional total‐score assessment. Frequency, centrality, bridge centrality and simulated perturbation response address different managerial questions. Low‐frequency but highly central activities such as vital sign monitoring may indicate broader disruption when missed and warrant protection through reliable surveillance routines, escalation procedures and documentation systems. Bridge activities such as pressure ulcer/wound/ostomy care, rehabilitation guidance, discharge planning/teaching and dietary guidance may benefit from coordination across assessment, basic care, planning and patient education. Bathing/skin care and emotional support, which showed the most consistent alleviating rankings across models and doses, are reasonable candidates for carefully designed pilot interventions. Possible strategies include explicit allocation of responsibility for turning and skin care, structured rounding that covers both monitoring and comfort needs, rapid redistribution of workload during patient surges and communication support following workplace violence. These approaches should be evaluated prospectively rather than adopted on the assumption that a simulated node response represents an intervention effect. The more model‐sensitive aggravating rankings should not be used alone to determine quality‐improvement priorities.

Future research should examine both care activities and the organisational conditions under which they are missed. Moderated and multigroup network analyses could test whether working hours, staffing, patient acuity, workplace violence, department or professional characteristics alter particular edges, centrality rankings or bridge relationships. Multilevel models are needed to separate nurse‐level associations from differences among hospitals, whereas longitudinal networks could examine how changes in workload and work environment relate to subsequent changes in missed‐care patterns. Candidate strategies identified by the present simulations should then be evaluated in staged studies, beginning with feasibility and process evaluation and progressing to controlled longitudinal or cluster‐randomised designs. Replication across nontertiary hospitals, other clinical specialties, different regions and international healthcare systems will also be necessary before the observed network topology or node rankings can be considered broadly transferable.

### 4.5. Limitations

Several limitations should be acknowledged. First, the cross‐sectional design precludes temporal or causal inference, and the NIRA findings represent model‐implied responses to hypothetical threshold perturbations rather than observed intervention effects. Although common‐offset recalibration aligned the model‐implied mean number of active nodes with the observed mean, it did not fully reproduce the observed total‐score dispersion or the probabilities of extreme scores. The estimated perturbation magnitudes and node rankings therefore remain conditional on the fitted Ising distribution and may have been affected by this residual model misfit. Longitudinal observational studies and prospective intervention studies are needed to determine whether interventions designed to reduce omission of the identified care activities alter subsequent patterns of missed nursing care. Second, dichotomisation collapsed responses from ‘rarely missed’ to ‘always missed’ into a single category, removing information about the original frequency gradient. This recoding may have affected node distributions, edge selection and edge weights, centrality and bridge centrality estimates and NIRA rankings. The GGM based on the original ordered responses showed strong agreement with the Ising network in the key BEI findings, but this sensitivity analysis cannot recover the information removed from the Ising and NIRA analyses or demonstrate that all network estimates were unaffected by dichotomisation. The four node communities were defined according to the MISSCARE care‐activity classification to support network interpretation and bridge centrality analysis and should not be interpreted as validated subscales of the Chinese MISSCARE Survey. Third, missed nursing care was self‐reported and may have been affected by recall and social desirability bias. Future studies could combine self‐report measures with clinical audits, direct observation or electronic nursing records. Fourth, the total numbers of eligible nurses and nurses invited to participate across the 22 hospitals were unavailable; consequently, an individual‐level response rate could not be calculated. Differences between participating and nonparticipating eligible nurses could not be assessed, creating the possibility of nonresponse and selection bias. The analytic sample may therefore not fully represent all eligible nurses at the participating hospitals, and the estimated item occurrence proportions and network characteristics may have been affected. Fifth, hospital identifiers were entered into a free‐text field and could not be mapped reliably to the 22 participating hospitals for all individual records. Consequently, hospital‐level clustering could not be quantified, and cluster‐aware resampling or hospital‐adjusted sensitivity analyses could not be conducted. The pooled network and NIRA estimates may reflect both within‐hospital associations and between‐hospital differences. Participant‐level bootstrap intervals may therefore be too narrow, and the CS coefficients may overstate centrality stability. Future multicentre studies should use predefined hospital codes or site‐specific survey links to support multilevel network analysis and hospital‐level resampling. Sixth, although hospitals were selected from different geographical strata within the province, all participating institutions were tertiary Grade A hospitals in a single province. The findings may therefore not generalise to nontertiary hospitals, other nursing specialties or healthcare systems with different organisational and resource conditions. Replication in geographically and internationally diverse samples from different hospital tiers, together with adequately powered subgroup comparisons by professional title, department, workload and other occupational characteristics, is needed to assess the consistency of the network topology, central and bridge nodes and NIRA rankings.

### 4.6. Conclusion

The item‐level network of missed nursing care among emergency and critical care nurses was predominantly positive. Vital sign monitoring ranked highest for EI, whereas pressure ulcer/wound/ostomy care, rehabilitation guidance, discharge planning/teaching, emotional support and dietary guidance were the five highest‐ranked nodes for BEI. In the primary calibrated original eLasso model, assessment and planning activities were prominent under aggravating threshold perturbations, whereas emotional support and several basic‐care activities were prominent under alleviating threshold perturbations. Across all examined doses and both model parameterisations, bathing/skin care and emotional support were the only activities that remained among the five highest‐ranked alleviating nodes. These findings indicate that item occurrence, centrality, bridge connectivity and responsiveness to model‐based perturbation capture distinct aspects of missed nursing care. The NIRA findings remain conditional on the fitted Ising models and are hypothesis‐generating rather than evidence of intervention effects; they highlight candidate activities for further evaluation in longitudinal observational studies and prospective intervention studies.

## Author Contributions

Fanfan Lv: writing–reviewing and editing, supervision, methodology, data curation and conceptualisation.

Na Wang: visualisation, supervision, investigation and data curation.

Yan Cao: validation and supervision.

Jia Jia: writing–reviewing and editing, supervision, resources, project administration, methodology, investigation, data curation and conceptualisation.

## Funding

This study was supported by the Key Philosophy and Social Science Research Project of Department of Education of Hubei Province (Grant/Award Number: 24D088).

## Ethics Statement

Ethical approval was obtained from the Medical Ethics Committee of Shiyan Taihe Affiliated Hospital of Hubei University of Medicine (approval no. 2024KS53).

## Conflicts of Interest

The authors declare no conflicts of interest.

## Supporting Information

Additional supporting information can be found online in the Supporting Information section.

## Supporting information


**Supporting Information** The supporting information includes additional tables and figures reporting the original item‐response distributions, bootstrap analyses, model‐calibration diagnostics, moderation analysis, permutation validation, repeated‐simulation stability, dose sensitivity, model sensitivity and GGM sensitivity analysis. The complete annotated R code for the NIRA threshold‐perturbation analyses is also provided to support reproducibility.

## Data Availability

The data that support the findings of this study are available from the corresponding author upon reasonable request.
