# Supplementary material for: Missed Nursing Care Among Emergency and Critical Care Nurses: An Item‐Level Network Analysis With Computer‐Simulated Interventions
Source: J Nurs Manag. 2026 Sep 27;2026:5019343. doi: 10.1155/jonm/5019343 (PMC13616138; doi:10.1155/jonm/5019343)
Supplement: Supplementary file 1 [file JONM-2026-5019343-s001.zip › Supplementary_R_Code_NIRA .docx]

\# =============================================================================

\# Supplementary R code

\# Common-offset mean recalibration, exact state enumeration, and

\# NodeIdentifyR threshold-perturbation analysis

\# =============================================================================

\#

\# Required input

\#	The final de-identified analytic CSV used for the reported sample of 3,351

\#	nurses, containing the 24 MISSCARE care-activity items

\#	named A1, A2, ..., A24. Each item must be coded from 0 to 4, where 0 denotes

\#	"never missed" and 1-4 denote increasing reported frequencies of missed

\#	care. The analysis dichotomises these responses as 0 versus 1-4.

\#

\# Scope

\#	1. estimation of the original eLasso Ising model;

\#	2. unpenalised post-selection refitting on the eLasso-selected topology;

\#	3. common-offset mean recalibration of both parameterisations;

\#	4. exact enumeration of all 2^24 binary states;

\#	5. exact single-node threshold perturbations at 0.5, 1.0, 1.5, and 2.0

\#	model-specific threshold standard deviations in both directions;

\#	6. comparison of the primary original eLasso model with the

\#	post-selection sensitivity model;

\#	7. moderation assessment with 1,000 resamples per candidate moderator;

\#	8. permutation validation using 5,000 model-implied total scores per

\#	condition and 5,000 two-sided permutations; and

\#	9. repeated-simulation ranking stability across 1,000 repetitions, with

\#	5,000 model-implied total scores per condition in each repetition.

\#

\# The exact point estimates and rankings do not use Monte Carlo averages.

\# Simulation is used only for the permutation and repeated-simulation

\# validation procedures.

\#

\# Software used for the reported analysis

\#	R 4.5.2

\#	IsingFit 0.4

\#	IsingSampler 0.2.4

\#	mgm 1.2-15

\#

\# Important

\#	The output refers to model-implied changes in the number of dichotomised

\#	active nodes. It does not estimate changes in the original 0-4 MISSCARE

\#	total score or causal intervention effects.

\# =============================================================================

graphics.off()

\# -----------------------------------------------------------------------------

\# 1. Required R packages

\# -----------------------------------------------------------------------------

required_packages <- c(

"IsingFit", # version 0.4 in the reported analysis

"IsingSampler", # version 0.2.4 in the reported analysis

"mgm"	# version 1.2-15 in the reported analysis

)

\# If necessary, install the packages once before running the analysis:

\# install.packages(required_packages)

missing_packages <- required_packages[

!vapply(required_packages, requireNamespace, logical(1), quietly = TRUE)

]

if (length(missing_packages) > 0L) {

stop(

"The following required R packages are not installed: ",

paste(missing_packages, collapse = ", "),

". Install them before running this script."

)

}

suppressPackageStartupMessages(

invisible(lapply(required_packages, library, character.only = TRUE))

)

if (utils::packageVersion("mgm") < "1.2.0") {

stop("mgm version 1.2.0 or later is required.")

}

\# -----------------------------------------------------------------------------

\# 2. Input data file and output directory

\# -----------------------------------------------------------------------------

\# The reported analysis used the final de-identified dataset of 3,351 nurses.

\# The CSV must contain columns A1-A24, coded 0-4. Extra columns are ignored.

DATA_FILE <- "MNC_analysis_data.csv"

OUTPUT_DIR <- "NIRA_reproducibility_output"

command_line_arguments <- commandArgs(trailingOnly = TRUE)

if (length(command_line_arguments) >= 1L) {

DATA_FILE <- command_line_arguments[1L]

}

if (length(command_line_arguments) >= 2L) {

OUTPUT_DIR <- command_line_arguments[2L]

}

if (!file.exists(DATA_FILE) && interactive()) {

message(

"Default input file not found: ", DATA_FILE, ". Select the final ",

"de-identified analytic CSV file in the file-selection window."

)

selected_data_file <- tryCatch(

file.choose(),

error = function(error_condition) ""

)

if (nzchar(selected_data_file)) {

DATA_FILE <- selected_data_file

}

}

if (!file.exists(DATA_FILE)) {

stop(

"Input file not found: ", DATA_FILE,

". Edit DATA_FILE or supply the input path as the first command-line ",

"argument."

)

}

dir.create(OUTPUT_DIR, showWarnings = FALSE, recursive = TRUE)

\# -----------------------------------------------------------------------------

\# 3. MISSCARE items and prespecified communities

\# -----------------------------------------------------------------------------

\# These are the exact 24 variables extracted from the selected CSV file.

node_labels <- paste0("A", seq_len(24L))

node_names <- unname(c(

A1 = "Bed linen care",

A2 = "Turning every 2 hours",

A3 = "Dietary guidance",

A4 = "Mouth care",

A5 = "Bathing/skin care",

A6 = "Toileting assistance",

A7 = "Emotional support",

A8 = "PRN medication",

A9 = "Timely medication administration",

A10 = "Call light response",

A11 = "Pressure ulcer/wound/ostomy care",

A12 = "Health education",

A13 = "Rehabilitation guidance",

A14 = "Focused reassessment/care-plan updating",

A15 = "IV/drainage tube care",

A16 = "Risk assessment",

A17 = "Intake/output monitoring",

A18 = "Vital sign monitoring",

A19 = "Bedside glucose monitoring",

A20 = "Nursing documentation",

A21 = "Medical record review",

A22 = "Ward rounds",

A23 = "Discharge planning/teaching",

A24 = "Hand hygiene"

))

communities <- unname(c(

A1 = "Basic care",

A2 = "Basic care",

A3 = "Planning",

A4 = "Basic care",

A5 = "Basic care",

A6 = "Individual needs",

A7 = "Individual needs",

A8 = "Individual needs",

A9 = "Individual needs",

A10 = "Individual needs",

A11 = "Basic care",

A12 = "Planning",

A13 = "Planning",

A14 = "Assessment",

A15 = "Assessment",

A16 = "Assessment",

A17 = "Assessment",

A18 = "Assessment",

A19 = "Assessment",

A20 = "Assessment",

A21 = "Assessment",

A22 = "Assessment",

A23 = "Planning",

A24 = "Basic care"

))

if (

length(node_names) != length(node_labels) ||

length(communities) != length(node_labels) ||

anyNA(node_names) ||

anyNA(communities)

) {

stop("The 24-item specification is incomplete.")

}

item_dictionary <- data.frame(

Node = node_labels,

Item = node_names,

Community = communities,

stringsAsFactors = FALSE

)

\# -----------------------------------------------------------------------------

\# 4. Analysis settings

\# -----------------------------------------------------------------------------

ELASSO_EBIC_GAMMA <- 0.25

ELASSO_AND_RULE <- TRUE

EXACT_BLOCK_SIZE <- 256L

DOSES_SD <- c(0.5, 1.0, 1.5, 2.0)

PRIMARY_DOSE_SD <- 2.0

TOP_K <- 5L

RUN_MODERATION <- TRUE

MODERATION_EBIC_GAMMA <- 0.25

MODERATION_RULE <- "AND"

MODERATION_RESAMPLES <- 1000L

MODERATION_QUANTILES <- c(0.025, 0.975)

REUSE_MODERATION_CHECKPOINTS <- TRUE

N_SIMULATED_SCORES <- 5000L

N_PERMUTATIONS <- 5000L

PERMUTATION_BATCH_SIZE <- 500L

N_STABILITY_REPETITIONS <- 1000L

RANDOM_SEED <- 20260820L

REPORTED_SAMPLE_SIZE <- 3351L

if (

length(DOSES_SD) == 0L ||

any(!is.finite(DOSES_SD)) ||

any(DOSES_SD <= 0) ||

anyDuplicated(DOSES_SD)

) {

stop("DOSES_SD must contain distinct, finite, positive values.")

}

if (!any(abs(DOSES_SD - PRIMARY_DOSE_SD) < 1e-12)) {

stop("PRIMARY_DOSE_SD must be included in DOSES_SD.")

}

if (

EXACT_BLOCK_SIZE < 1L ||

N_SIMULATED_SCORES < 2L ||

N_PERMUTATIONS < 1L ||

PERMUTATION_BATCH_SIZE < 1L ||

N_STABILITY_REPETITIONS < 1L ||

MODERATION_RESAMPLES < 1L

) {

stop("One or more analysis counts are invalid.")

}

\# -----------------------------------------------------------------------------

\# 5. Read, extract, validate, and dichotomise the 24 MISSCARE items

\# -----------------------------------------------------------------------------

raw_data <- utils::read.csv(

DATA_FILE,

header = TRUE,

check.names = FALSE,

stringsAsFactors = FALSE

)

missing_item_columns <- setdiff(node_labels, names(raw_data))

if (length(missing_item_columns) > 0L) {

stop(

"The input file is missing the following required columns: ",

paste(missing_item_columns, collapse = ", "), "."

)

}

\# Retain A1-A24 in their prespecified order; all other CSV columns are excluded.

ordered_data <- raw_data[, node_labels, drop = FALSE]

for (item_index in seq_along(ordered_data)) {

original_values <- ordered_data[[item_index]]

if (is.factor(original_values)) {

original_values <- as.character(original_values)

}

numeric_values <- suppressWarnings(as.numeric(original_values))

newly_missing <- is.na(numeric_values) & !is.na(original_values)

if (any(newly_missing)) {

stop(

"Non-numeric values were found in ", node_labels[item_index], "."

)

}

ordered_data[[item_index]] <- numeric_values

}

if (anyNA(ordered_data)) {

stop(

"Missing item responses were detected. The reported analysis contained ",

"no missing responses and did not use imputation or complete-case deletion."

)

}

ordered_matrix <- as.matrix(ordered_data)

storage.mode(ordered_matrix) <- "double"

if (!all(ordered_matrix %in% 0:4)) {

stop("All A1-A24 responses must be integers from 0 to 4.")

}

binary_matrix <- 1L * (ordered_matrix >= 1)

storage.mode(binary_matrix) <- "integer"

colnames(binary_matrix) <- node_labels

if (any(vapply(

seq_len(ncol(binary_matrix)),

function(column_index) {

length(unique(binary_matrix[, column_index])) < 2L

},

logical(1)

))) {

stop("At least one dichotomised item has zero variance.")

}

n_observations <- nrow(binary_matrix)

n_nodes <- ncol(binary_matrix)

if (n_nodes != 24L) {

stop("This supplementary analysis is specified for exactly 24 nodes.")

}

if (n_observations != REPORTED_SAMPLE_SIZE) {

warning(

"The reported analysis used ", REPORTED_SAMPLE_SIZE,

" participants, whereas the selected input file contains ",

n_observations, "."

)

}

message("Input dataset: ", normalizePath(DATA_FILE, mustWork = TRUE))

message("Participants read: ", n_observations)

message("MISSCARE variables retained: ", paste(node_labels, collapse = ", "))

observed_node_probability <- colMeans(binary_matrix)

observed_sum_score <- rowSums(binary_matrix)

observed_mean_sum_score <- mean(observed_sum_score)

observed_mean_activation <- observed_mean_sum_score / n_nodes

score_values <- 0:n_nodes

observed_score_probability <- tabulate(

observed_sum_score + 1L,

nbins = n_nodes + 1L

) / n_observations

\# -----------------------------------------------------------------------------

\# 6. Original eLasso model and post-selection sensitivity model

\# -----------------------------------------------------------------------------

standardise_weight_matrix <- function(weight_matrix, model_name) {

weight_matrix <- as.matrix(weight_matrix)

storage.mode(weight_matrix) <- "double"

if (!all(dim(weight_matrix) == c(n_nodes, n_nodes))) {

stop(model_name, " returned a weight matrix with unexpected dimensions.")

}

if (any(!is.finite(weight_matrix))) {

stop(model_name, " returned non-finite interaction parameters.")

}

weight_matrix <- (weight_matrix + t(weight_matrix)) / 2

diag(weight_matrix) <- 0

rownames(weight_matrix) <- colnames(weight_matrix) <- node_labels

weight_matrix

}

standardise_threshold_vector <- function(threshold_vector, model_name) {

threshold_vector <- as.numeric(threshold_vector)

if (length(threshold_vector) != n_nodes) {

stop(model_name, " returned an unexpected number of thresholds.")

}

if (any(!is.finite(threshold_vector))) {

stop(model_name, " returned non-finite thresholds.")

}

names(threshold_vector) <- node_labels

threshold_vector

}

message("Estimating the original eLasso Ising model.")

elasso_fit <- IsingFit::IsingFit(

data = as.data.frame(binary_matrix),

gamma = ELASSO_EBIC_GAMMA,

AND = ELASSO_AND_RULE,

plot = FALSE,

progressbar = TRUE

)

weight_elasso <- standardise_weight_matrix(

elasso_fit$weiadj,

"Original eLasso model"

)

threshold_elasso <- standardise_threshold_vector(

elasso_fit$thresholds,

"Original eLasso model"

)

selected_adjacency <- 1L * (

abs(weight_elasso) > 0 & abs(t(weight_elasso)) > 0

)

diag(selected_adjacency) <- 0L

rownames(selected_adjacency) <- colnames(selected_adjacency) <- node_labels

if (sum(selected_adjacency[upper.tri(selected_adjacency)]) == 0L) {

stop("The eLasso model retained no edges; post-selection refitting stopped.")

}

message("Estimating the unpenalised post-selection sensitivity model.")

set.seed(20260815L)

post_selection_fit <- IsingSampler::EstimateIsing(

data = binary_matrix,

responses = c(0L, 1L),

beta = 1,

method = "uni",

adj = selected_adjacency

)

weight_post_selection <- standardise_weight_matrix(

post_selection_fit$graph,

"Post-selection model"

)

weight_post_selection[selected_adjacency == 0L] <- 0

diag(weight_post_selection) <- 0

threshold_post_selection <- standardise_threshold_vector(

post_selection_fit$thresholds,

"Post-selection model"

)

utils::write.csv(

weight_elasso,

file.path(OUTPUT_DIR, "01_primary_elasso_interaction_matrix.csv"),

row.names = TRUE

)

utils::write.csv(

weight_post_selection,

file.path(OUTPUT_DIR, "02_sensitivity_post_selection_interaction_matrix.csv"),

row.names = TRUE

)

\# -----------------------------------------------------------------------------

\# 7. Exact 2^24-state enumeration and common-offset mean recalibration

\# -----------------------------------------------------------------------------

\# With binary responses x in {0,1}, the joint probability kernel used here is

\# proportional to

\#	exp(sum_i threshold_i*x_i + sum_{i<j} weight_ij*x_i*x_j).

\# A common offset c multiplies the mass of every state with sum score s by

\# exp(c*s). Consequently, c can be obtained from the exact score masses while

\# leaving every interaction parameter and every between-node threshold

\# difference unchanged.

make_binary_states <- function(number_of_bits) {

number_of_states <- as.integer(2^number_of_bits)

state_ids <- as.integer(0:(number_of_states - 1L))

state_matrix <- vapply(

0:(number_of_bits - 1L),

function(bit_index) {

as.integer(bitwAnd(bitwShiftR(state_ids, bit_index), 1L))

},

integer(number_of_states)

)

state_matrix <- matrix(

state_matrix,

nrow = number_of_states,

ncol = number_of_bits

)

storage.mode(state_matrix) <- "double"

state_matrix

}

internal_energy <- function(states, thresholds, within_weights) {

as.vector(states %*% thresholds) +

0.5 * rowSums((states %*% within_weights) * states)

}

probability_from_score_mass <- function(score_mass, common_offset = 0) {

scores <- 0:(length(score_mass) - 1L)

if (any(!is.finite(score_mass)) || any(score_mass <= 0)) {

stop("Exact score masses must be finite and strictly positive.")

}

log_probability <- log(score_mass) + common_offset * scores

log_probability <- log_probability - max(log_probability)

probability <- exp(log_probability)

probability / sum(probability)

}

n_left <- floor(n_nodes / 2L)

n_right <- n_nodes - n_left

left_indices <- seq_len(n_left)

right_indices <- (n_left + 1L):n_nodes

left_states <- make_binary_states(n_left)

right_states <- make_binary_states(n_right)

left_scores <- rowSums(left_states)

right_scores <- rowSums(right_states)

right_score_indicator <- vapply(

0:n_right,

function(current_score) as.numeric(right_scores == current_score),

numeric(nrow(right_states))

)

right_score_indicator <- matrix(

right_score_indicator,

nrow = nrow(right_states),

ncol = n_right + 1L

)

right_score_rows <- lapply(

0:n_right,

function(current_score) which(right_scores == current_score)

)

left_block_starts <- seq.int(

1L,

nrow(left_states),

by = EXACT_BLOCK_SIZE

)

enumerate_uncalibrated_masses <- function(

weight_matrix,

threshold_vector,

model_name

) {

message(

model_name, ": enumerating ",

format(2^n_nodes, scientific = FALSE, big.mark = ","),

" binary states."

)

weight_left <- weight_matrix[

left_indices,

left_indices,

drop = FALSE

]

weight_right <- weight_matrix[

right_indices,

right_indices,

drop = FALSE

]

weight_cross <- weight_matrix[

left_indices,

right_indices,

drop = FALSE

]

energy_left <- internal_energy(

left_states,

threshold_vector[left_indices],

weight_left

)

energy_right <- internal_energy(

right_states,

threshold_vector[right_indices],

weight_right

)

right_cross_projection <- weight_cross %*% t(right_states)

score_mass <- numeric(n_nodes + 1L)

node_score_active_mass <- matrix(

0,

nrow = n_nodes,

ncol = n_nodes + 1L,

dimnames = list(node_labels, as.character(0:n_nodes))

)

running_maximum <- -Inf

for (block_number in seq_along(left_block_starts)) {

first_row <- left_block_starts[block_number]

last_row <- min(

first_row + EXACT_BLOCK_SIZE - 1L,

nrow(left_states)

)

block_rows <- first_row:last_row

block_left_scores <- left_scores[block_rows]

log_weight <- left_states[block_rows, , drop = FALSE] %*%

right_cross_projection

log_weight <- sweep(log_weight, 1L, energy_left[block_rows], "+")

log_weight <- sweep(log_weight, 2L, energy_right, "+")

block_maximum <- max(log_weight)

updated_maximum <- max(running_maximum, block_maximum)

if (is.finite(running_maximum) && updated_maximum > running_maximum) {

rescaling_factor <- exp(running_maximum - updated_maximum)

score_mass <- score_mass * rescaling_factor

node_score_active_mass <-

node_score_active_mass * rescaling_factor

}

block_weight <- exp(log_weight - updated_maximum)

mass_by_right_score <- block_weight %*% right_score_indicator

for (local_row in seq_along(block_rows)) {

total_scores <- block_left_scores[local_row] + 0:n_right

score_slots <- total_scores + 1L

row_score_mass <- mass_by_right_score[local_row, ]

score_mass[score_slots] <-

score_mass[score_slots] + row_score_mass

active_left_nodes <- which(

left_states[block_rows[local_row], ] > 0.5

)

if (length(active_left_nodes) > 0L) {

for (left_node in active_left_nodes) {

node_score_active_mass[left_node, score_slots] <-

node_score_active_mass[left_node, score_slots] + row_score_mass

}

}

}

for (left_score_value in sort(unique(block_left_scores))) {

local_left_rows <- which(block_left_scores == left_score_value)

right_state_mass <- colSums(

block_weight[local_left_rows, , drop = FALSE]

)

for (right_score_value in 0:n_right) {

selected_right_rows <- right_score_rows[[right_score_value + 1L]]

total_score <- left_score_value + right_score_value

right_node_mass <- as.vector(crossprod(

right_state_mass[selected_right_rows],

right_states[selected_right_rows, , drop = FALSE]

))

node_score_active_mass[

right_indices,

total_score + 1L

] <- node_score_active_mass[

right_indices,

total_score + 1L

] + right_node_mass

}

}

running_maximum <- updated_maximum

if (

block_number %% 4L == 0L ||

block_number == length(left_block_starts)

) {

message(

" exact-enumeration block ", block_number, "/",

length(left_block_starts)

)

}

}

if (any(score_mass <= 0) || any(!is.finite(score_mass))) {

stop(model_name, ": invalid exact score masses were obtained.")

}

list(

score_mass = score_mass,

node_score_active_mass = node_score_active_mass

)

}

calibrate_exact_model <- function(

weight_matrix,

threshold_vector,

model_role,

model_name

) {

uncalibrated_masses <- enumerate_uncalibrated_masses(

weight_matrix,

threshold_vector,

model_name

)

uncalibrated_score_probability <- probability_from_score_mass(

uncalibrated_masses$score_mass,

common_offset = 0

)

uncalibrated_mean_sum_score <- sum(

score_values * uncalibrated_score_probability

)

mean_difference <- function(common_offset) {

shifted_probability <- probability_from_score_mass(

uncalibrated_masses$score_mass,

common_offset

)

sum(score_values * shifted_probability) - observed_mean_sum_score

}

if (mean_difference(-20) > 0 || mean_difference(20) < 0) {

stop(

model_name,

": the observed mean was not bracketed by common offsets from -20 to 20."

)

}

common_offset <- stats::uniroot(

mean_difference,

interval = c(-20, 20),

tol = 1e-11

)$root

offset_log_multiplier <- common_offset * score_values

offset_multiplier <- exp(

offset_log_multiplier - max(offset_log_multiplier)

)

calibrated_score_mass <-

uncalibrated_masses$score_mass * offset_multiplier

calibrated_node_score_active_mass <- sweep(

uncalibrated_masses$node_score_active_mass,

2L,

offset_multiplier,

"*"

)

total_mass <- sum(calibrated_score_mass)

calibrated_score_probability <- calibrated_score_mass / total_mass

node_score_joint_probability <-

calibrated_node_score_active_mass / total_mass

calibrated_node_probability <- rowSums(node_score_joint_probability)

calibrated_mean_sum_score <- sum(

score_values * calibrated_score_probability

)

identity_error <- max(abs(

colSums(node_score_joint_probability) -

score_values * calibrated_score_probability

))

if (!is.finite(identity_error) || identity_error > 1e-10) {

stop(model_name, ": the exact-enumeration identity check failed.")

}

if (abs(calibrated_mean_sum_score - observed_mean_sum_score) > 1e-8) {

stop(model_name, ": common-offset mean recalibration did not converge.")

}

calibrated_thresholds <- threshold_vector + common_offset

names(calibrated_thresholds) <- node_labels

names(calibrated_node_probability) <- node_labels

message(

model_name,

": uncalibrated mean sum score = ",

sprintf("%.6f", uncalibrated_mean_sum_score),

"; common offset = ", sprintf("%+.9f", common_offset),

"; recalibrated mean sum score = ",

sprintf("%.6f", calibrated_mean_sum_score), "."

)

list(

role = model_role,

name = model_name,

weight_matrix = weight_matrix,

base_thresholds = threshold_vector,

common_offset = common_offset,

calibrated_thresholds = calibrated_thresholds,

threshold_sd = stats::sd(calibrated_thresholds),

uncalibrated_mean_sum_score = uncalibrated_mean_sum_score,

uncalibrated_mean_activation =

uncalibrated_mean_sum_score / n_nodes,

score_mass = calibrated_score_mass,

node_score_active_mass = calibrated_node_score_active_mass,

score_probability = calibrated_score_probability,

node_probability = calibrated_node_probability,

mean_sum_score = calibrated_mean_sum_score,

mean_activation = calibrated_mean_sum_score / n_nodes,

exact_state_count = 2^n_nodes,

identity_error = identity_error

)

}

\# The final manuscript roles are fixed here. The original eLasso model is the

\# primary NIRA model; the unpenalised post-selection refit is the sensitivity

\# model.

primary_model <- calibrate_exact_model(

weight_matrix = weight_elasso,

threshold_vector = threshold_elasso,

model_role = "Primary",

model_name = "Original eLasso with common-offset mean recalibration"

)

invisible(gc())

sensitivity_model <- calibrate_exact_model(

weight_matrix = weight_post_selection,

threshold_vector = threshold_post_selection,

model_role = "Sensitivity",

model_name = paste0(

"Unpenalised post-selection refit with common-offset mean recalibration"

)

)

invisible(gc())

\# -----------------------------------------------------------------------------

\# 8. Calibration diagnostics

\# -----------------------------------------------------------------------------

safe_correlation <- function(first_vector, second_vector) {

if (

stats::sd(first_vector) == 0 ||

stats::sd(second_vector) == 0

) {

return(NA_real_)

}

stats::cor(first_vector, second_vector, method = "pearson")

}

distribution_moments <- function(probability) {

distribution_mean <- sum(score_values * probability)

distribution_variance <- sum(

probability * (score_values - distribution_mean)^2

)

c(

Mean = distribution_mean,

SD = sqrt(max(0, distribution_variance))

)

}

calibration_summary_row <- function(model_object) {

node_error <- model_object$node_probability - observed_node_probability

observed_moments <- distribution_moments(observed_score_probability)

model_moments <- distribution_moments(model_object$score_probability)

data.frame(

ModelRole = model_object$role,

Model = model_object$name,

NumberOfParticipants = n_observations,

NumberOfNodes = n_nodes,

ExactStateCount = model_object$exact_state_count,

UncalibratedMeanSumScore = model_object$uncalibrated_mean_sum_score,

UncalibratedMeanActivationProportion =

model_object$uncalibrated_mean_activation,

CommonOffsetC = model_object$common_offset,

RecalibratedMeanSumScore = model_object$mean_sum_score,

RecalibratedMeanActivationProportion = model_object$mean_activation,

ObservedMeanSumScore = observed_mean_sum_score,

ObservedMeanActivationProportion = observed_mean_activation,

NodeProbabilityPearsonCorrelation = safe_correlation(

observed_node_probability,

model_object$node_probability

),

NodeProbabilityMeanAbsoluteError = mean(abs(node_error)),

NodeProbabilityMeanAbsoluteErrorPercentagePoints =

100 * mean(abs(node_error)),

NodeProbabilityMaximumAbsoluteError = max(abs(node_error)),

NodeProbabilityMaximumAbsoluteErrorPercentagePoints =

100 * max(abs(node_error)),

ObservedSumScoreSD = unname(observed_moments["SD"]),

ModelImpliedSumScoreSD = unname(model_moments["SD"]),

ObservedProbabilityScore0 = observed_score_probability[1L],

ModelImpliedProbabilityScore0 = model_object$score_probability[1L],

ObservedProbabilityScore24 =

observed_score_probability[n_nodes + 1L],

ModelImpliedProbabilityScore24 =

model_object$score_probability[n_nodes + 1L],

TotalVariationDistance = 0.5 * sum(abs(

model_object$score_probability - observed_score_probability

)),

KolmogorovSmirnovDistance = max(abs(

cumsum(model_object$score_probability) -

cumsum(observed_score_probability)

)),

ExactEnumerationIdentityError = model_object$identity_error,

stringsAsFactors = FALSE

)

}

calibration_summary <- rbind(

calibration_summary_row(primary_model),

calibration_summary_row(sensitivity_model)

)

calibration_node_probabilities <- rbind(

data.frame(

ModelRole = primary_model$role,

Model = primary_model$name,

Node = node_labels,

Item = node_names,

Community = communities,

ObservedProbability = observed_node_probability,

ModelImpliedProbability = primary_model$node_probability,

Error = primary_model$node_probability - observed_node_probability,

AbsoluteError = abs(

primary_model$node_probability - observed_node_probability

),

stringsAsFactors = FALSE

),

data.frame(

ModelRole = sensitivity_model$role,

Model = sensitivity_model$name,

Node = node_labels,

Item = node_names,

Community = communities,

ObservedProbability = observed_node_probability,

ModelImpliedProbability = sensitivity_model$node_probability,

Error = sensitivity_model$node_probability - observed_node_probability,

AbsoluteError = abs(

sensitivity_model$node_probability - observed_node_probability

),

stringsAsFactors = FALSE

)

)

calibration_score_distributions <- rbind(

data.frame(

ModelRole = "Observed",

Model = "Observed data",

Score = score_values,

Probability = observed_score_probability,

stringsAsFactors = FALSE

),

data.frame(

ModelRole = primary_model$role,

Model = primary_model$name,

Score = score_values,

Probability = primary_model$score_probability,

stringsAsFactors = FALSE

),

data.frame(

ModelRole = sensitivity_model$role,

Model = sensitivity_model$name,

Score = score_values,

Probability = sensitivity_model$score_probability,

stringsAsFactors = FALSE

)

)

calibrated_thresholds <- rbind(

data.frame(

ModelRole = primary_model$role,

Model = primary_model$name,

Node = node_labels,

Item = node_names,

BaseThreshold = primary_model$base_thresholds,

CommonOffsetC = primary_model$common_offset,

RecalibratedThreshold = primary_model$calibrated_thresholds,

ThresholdSD = primary_model$threshold_sd,

stringsAsFactors = FALSE

),

data.frame(

ModelRole = sensitivity_model$role,

Model = sensitivity_model$name,

Node = node_labels,

Item = node_names,

BaseThreshold = sensitivity_model$base_thresholds,

CommonOffsetC = sensitivity_model$common_offset,

RecalibratedThreshold = sensitivity_model$calibrated_thresholds,

ThresholdSD = sensitivity_model$threshold_sd,

stringsAsFactors = FALSE

)

)

utils::write.csv(

calibration_summary,

file.path(OUTPUT_DIR, "03_common_offset_calibration_summary.csv"),

row.names = FALSE

)

utils::write.csv(

calibration_node_probabilities,

file.path(OUTPUT_DIR, "04_observed_vs_model_node_probabilities.csv"),

row.names = FALSE

)

utils::write.csv(

calibration_score_distributions,

file.path(OUTPUT_DIR, "05_observed_vs_model_sum_score_distributions.csv"),

row.names = FALSE

)

utils::write.csv(

calibrated_thresholds,

file.path(OUTPUT_DIR, "06_recalibrated_thresholds.csv"),

row.names = FALSE

)

\# -----------------------------------------------------------------------------

\# 9. Exact threshold-perturbation analysis

\# -----------------------------------------------------------------------------

perturb_score_distribution <- function(

model_object,

target_index,

threshold_change

) {

perturbed_score_mass <- model_object$score_mass +

(exp(threshold_change) - 1) *

model_object$node_score_active_mass[target_index, ]

if (

any(!is.finite(perturbed_score_mass)) ||

any(perturbed_score_mass <= 0)

) {

stop("Invalid score mass after threshold perturbation.")

}

probability <- perturbed_score_mass / sum(perturbed_score_mass)

moments <- distribution_moments(probability)

target_active_mass <- exp(threshold_change) *

sum(model_object$node_score_active_mass[target_index, ])

list(

probability = probability,

mean_sum_score = unname(moments["Mean"]),

sd_sum_score = unname(moments["SD"]),

mean_activation = unname(moments["Mean"]) / n_nodes,

target_activation_probability =

target_active_mass / sum(perturbed_score_mass),

probability_score_0 = probability[1L],

probability_score_24 = probability[n_nodes + 1L]

)

}

run_exact_nira <- function(model_object, doses_sd) {

result_rows <- vector(

"list",

length(doses_sd) * 2L * n_nodes

)

distribution_rows <- vector(

"list",

1L + length(doses_sd) * 2L * n_nodes

)

distribution_rows[[1L]] <- data.frame(

ModelRole = model_object$role,

Model = model_object$name,

Scenario = "Baseline",

DoseSD = 0,

TargetNode = "Original",

TargetItem = "Original baseline",

Score = score_values,

Probability = model_object$score_probability,

stringsAsFactors = FALSE

)

row_index <- 0L

distribution_index <- 1L

scenario_table <- data.frame(

Scenario = c("Aggravating", "Alleviating"),

Direction = c(1, -1),

stringsAsFactors = FALSE

)

for (current_dose_sd in doses_sd) {

for (scenario_index in seq_len(nrow(scenario_table))) {

scenario_name <- scenario_table$Scenario[scenario_index]

threshold_change <- scenario_table$Direction[scenario_index] *

current_dose_sd * model_object$threshold_sd

for (target_index in seq_len(n_nodes)) {

row_index <- row_index + 1L

distribution_index <- distribution_index + 1L

perturbation <- perturb_score_distribution(

model_object,

target_index,

threshold_change

)

signed_change <-

perturbation$mean_sum_score - model_object$mean_sum_score

result_rows[[row_index]] <- data.frame(

ModelRole = model_object$role,

Model = model_object$name,

Scenario = scenario_name,

DoseSD = current_dose_sd,

Node = node_labels[target_index],

Item = node_names[target_index],

Community = communities[target_index],

ThresholdBefore = model_object$calibrated_thresholds[target_index],

ThresholdChange = threshold_change,

ThresholdAfter =

model_object$calibrated_thresholds[target_index] +

threshold_change,

BaselineMeanSumScore = model_object$mean_sum_score,

BaselineMeanActivationProportion = model_object$mean_activation,

PostPerturbationMeanSumScore = perturbation$mean_sum_score,

PostPerturbationMeanActivationProportion =

perturbation$mean_activation,

SignedMeanSumScoreChange = signed_change,

EffectMagnitude = abs(signed_change),

ActivationPercentagePointChange =

100 * signed_change / n_nodes,

BaselineTargetActivationProbability =

model_object$node_probability[target_index],

PostPerturbationTargetActivationProbability =

perturbation$target_activation_probability,

PostPerturbationSumScoreSD = perturbation$sd_sum_score,

PostPerturbationProbabilityScore0 =

perturbation$probability_score_0,

PostPerturbationProbabilityScore24 =

perturbation$probability_score_24,

stringsAsFactors = FALSE

)

distribution_rows[[distribution_index]] <- data.frame(

ModelRole = model_object$role,

Model = model_object$name,

Scenario = scenario_name,

DoseSD = current_dose_sd,

TargetNode = node_labels[target_index],

TargetItem = node_names[target_index],

Score = score_values,

Probability = perturbation$probability,

stringsAsFactors = FALSE

)

}

}

}

results <- do.call(rbind, result_rows)

distributions <- do.call(rbind, distribution_rows)

results$Rank <- ave(

results$EffectMagnitude,

results$Scenario,

results$DoseSD,

FUN = function(values) rank(-values, ties.method = "min")

)

results$Rank <- as.integer(results$Rank)

results$TopFive <- results$Rank <= TOP_K

if (any(

results$Scenario == "Aggravating" &

results$SignedMeanSumScoreChange < -1e-12

)) {

stop(model_object$name, ": an aggravating perturbation changed sign.")

}

if (any(

results$Scenario == "Alleviating" &

results$SignedMeanSumScoreChange > 1e-12

)) {

stop(model_object$name, ": an alleviating perturbation changed sign.")

}

results <- results[order(

results$DoseSD,

results$Scenario,

results$Rank,

results$Node

), ]

rownames(results) <- NULL

list(results = results, distributions = distributions)

}

message("Running exact threshold perturbations for the primary model.")

primary_nira <- run_exact_nira(primary_model, DOSES_SD)

message("Running exact threshold perturbations for the sensitivity model.")

sensitivity_nira <- run_exact_nira(sensitivity_model, DOSES_SD)

exact_nira_results <- rbind(

primary_nira$results,

sensitivity_nira$results

)

exact_score_distributions <- rbind(

primary_nira$distributions,

sensitivity_nira$distributions

)

primary_2sd_results <- primary_nira$results[

abs(primary_nira$results$DoseSD - PRIMARY_DOSE_SD) < 1e-12,

]

utils::write.csv(

exact_nira_results,

file.path(OUTPUT_DIR, "07_exact_NIRA_all_models_all_doses.csv"),

row.names = FALSE

)

utils::write.csv(

primary_2sd_results,

file.path(OUTPUT_DIR, "08_exact_NIRA_primary_elasso_2SD.csv"),

row.names = FALSE

)

utils::write.csv(

exact_score_distributions,

file.path(OUTPUT_DIR, "09_exact_NIRA_sum_score_distributions.csv"),

row.names = FALSE

)

\# -----------------------------------------------------------------------------

\# 10. Parameterisation and dose sensitivity summaries

\# -----------------------------------------------------------------------------

comparison_columns <- c(

"Scenario", "DoseSD", "Node", "Item", "Community",

"SignedMeanSumScoreChange", "EffectMagnitude", "Rank", "TopFive"

)

primary_comparison <- primary_nira$results[, comparison_columns]

sensitivity_comparison <- sensitivity_nira$results[, comparison_columns]

names(primary_comparison)[6:9] <- paste0(

"Primary_",

names(primary_comparison)[6:9]

)

names(sensitivity_comparison)[6:9] <- paste0(

"Sensitivity_",

names(sensitivity_comparison)[6:9]

)

parameterisation_comparison <- merge(

primary_comparison,

sensitivity_comparison,

by = c("Scenario", "DoseSD", "Node", "Item", "Community"),

all = TRUE,

sort = FALSE

)

parameterisation_comparison$AbsoluteRankDifference <- abs(

parameterisation_comparison$Primary_Rank -

parameterisation_comparison$Sensitivity_Rank

)

parameterisation_comparison$TopFiveInBothModels <-

parameterisation_comparison$Primary_TopFive &

parameterisation_comparison$Sensitivity_TopFive

parameterisation_comparison <- parameterisation_comparison[order(

parameterisation_comparison$DoseSD,

parameterisation_comparison$Scenario,

parameterisation_comparison$Primary_Rank,

parameterisation_comparison$Sensitivity_Rank

), ]

rownames(parameterisation_comparison) <- NULL

agreement_rows <- list()

agreement_index <- 0L

for (dose_sd in DOSES_SD) {

for (scenario_name in c("Aggravating", "Alleviating")) {

agreement_index <- agreement_index + 1L

current <- parameterisation_comparison[

abs(parameterisation_comparison$DoseSD - dose_sd) < 1e-12 &

parameterisation_comparison$Scenario == scenario_name,

]

primary_top <- current$Node[current$Primary_TopFive]

sensitivity_top <- current$Node[current$Sensitivity_TopFive]

shared_top <- intersect(primary_top, sensitivity_top)

top_union <- union(primary_top, sensitivity_top)

agreement_rows[[agreement_index]] <- data.frame(

Scenario = scenario_name,

DoseSD = dose_sd,

SpearmanEffectMagnitudeCorrelation = suppressWarnings(stats::cor(

current$Primary_EffectMagnitude,

current$Sensitivity_EffectMagnitude,

method = "spearman",

use = "complete.obs"

)),

TopFiveOverlapCount = length(shared_top),

TopFiveJaccard = if (length(top_union) == 0L) {

NA_real_

} else {

length(shared_top) / length(top_union)

},

SharedTopFiveNodes = paste(shared_top, collapse = "; "),

PrimaryTopFiveNodes = paste(primary_top, collapse = "; "),

SensitivityTopFiveNodes = paste(

sensitivity_top,

collapse = "; "

),

stringsAsFactors = FALSE

)

}

}

parameterisation_agreement <- do.call(rbind, agreement_rows)

cross_dose_rows <- list()

cross_dose_index <- 0L

for (scenario_name in c("Aggravating", "Alleviating")) {

for (node_index in seq_len(n_nodes)) {

cross_dose_index <- cross_dose_index + 1L

current <- parameterisation_comparison[

parameterisation_comparison$Scenario == scenario_name &

parameterisation_comparison$Node == node_labels[node_index],

]

current <- current[order(current$DoseSD), ]

cross_dose_rows[[cross_dose_index]] <- data.frame(

Scenario = scenario_name,

Node = node_labels[node_index],

Item = node_names[node_index],

Community = communities[node_index],

DosesEvaluated = nrow(current),

TopFiveInBothModelsAtEveryDose =

nrow(current) == length(DOSES_SD) &&

all(current$TopFiveInBothModels),

DosesTopFiveInBothModels = paste(

current$DoseSD[current$TopFiveInBothModels],

collapse = "; "

),

stringsAsFactors = FALSE

)

}

}

cross_dose_robustness <- do.call(rbind, cross_dose_rows)

utils::write.csv(

parameterisation_comparison,

file.path(OUTPUT_DIR, "10_parameterisation_sensitivity_by_node.csv"),

row.names = FALSE

)

utils::write.csv(

parameterisation_agreement,

file.path(OUTPUT_DIR, "11_parameterisation_agreement_summary.csv"),

row.names = FALSE

)

utils::write.csv(

cross_dose_robustness,

file.path(OUTPUT_DIR, "12_cross_dose_top_five_robustness.csv"),

row.names = FALSE

)

\# -----------------------------------------------------------------------------

\# 11. Permutation validation of the primary 2-SD analysis

\# -----------------------------------------------------------------------------

get_exact_score_probability <- function(

distribution_table,

model_role,

scenario,

target_node,

dose_sd

) {

selected <- distribution_table[

distribution_table$ModelRole == model_role &

distribution_table$Scenario == scenario &

distribution_table$TargetNode == target_node &

abs(distribution_table$DoseSD - dose_sd) < 1e-12,

]

selected <- selected[order(selected$Score), ]

if (

nrow(selected) != n_nodes + 1L ||

!identical(as.integer(selected$Score), 0:n_nodes)

) {

stop(

"An exact sum-score distribution is incomplete for ",

model_role, "/", scenario, "/", target_node, "."

)

}

probability <- as.numeric(selected$Probability)

if (any(!is.finite(probability)) || any(probability < -1e-12)) {

stop("An exact sum-score distribution contains invalid probabilities.")

}

probability[probability < 0] <- 0

probability / sum(probability)

}

sample_score_counts <- function(probability, sample_size) {

as.integer(stats::rmultinom(1L, sample_size, probability)[, 1L])

}

sample_moments_from_counts <- function(counts) {

sample_size <- sum(counts)

sample_mean <- sum(score_values * counts) / sample_size

sample_variance <- sum(

counts * (score_values - sample_mean)^2

) / (sample_size - 1)

c(

Mean = sample_mean,

SD = sqrt(max(0, sample_variance)),

SE = sqrt(max(0, sample_variance)) / sqrt(sample_size)

)

}

permutation_p_value_from_counts <- function(

baseline_counts,

post_counts,

number_of_permutations,

batch_size

) {

n_baseline <- sum(baseline_counts)

n_post <- sum(post_counts)

pooled_counts <- baseline_counts + post_counts

retained_categories <- pooled_counts > 0L

pooled_nonzero <- pooled_counts[retained_categories]

retained_scores <- score_values[retained_categories]

observed_difference <-

sum(score_values * post_counts) / n_post -

sum(score_values * baseline_counts) / n_baseline

extreme_count <- 0L

completed <- 0L

while (completed < number_of_permutations) {

current_batch <- min(

batch_size,

number_of_permutations - completed

)

random_tables <- stats::r2dtable(

current_batch,

r = c(n_baseline, n_post),

c = pooled_nonzero

)

permuted_differences <- vapply(

random_tables,

function(current_table) {

sum(retained_scores * current_table[2L, ]) / n_post -

sum(retained_scores * current_table[1L, ]) / n_baseline

},

numeric(1)

)

extreme_count <- extreme_count + sum(

abs(permuted_differences) >= abs(observed_difference) - 1e-12

)

completed <- completed + current_batch

}

\# Plus-one correction prevents an estimated permutation p value of zero.

(extreme_count + 1) / (number_of_permutations + 1)

}

run_permutation_scenario <- function(scenario_name, seed_offset) {

set.seed(RANDOM_SEED + seed_offset)

baseline_probability <- get_exact_score_probability(

exact_score_distributions,

model_role = "Primary",

scenario = "Baseline",

target_node = "Original",

dose_sd = 0

)

baseline_counts <- sample_score_counts(

baseline_probability,

N_SIMULATED_SCORES

)

baseline_sample_moments <- sample_moments_from_counts(baseline_counts)

exact_scenario <- primary_2sd_results[

primary_2sd_results$Scenario == scenario_name,

]

exact_scenario <- exact_scenario[order(exact_scenario$Rank), ]

output_rows <- vector("list", n_nodes)

for (row_index in seq_len(n_nodes)) {

target_node <- exact_scenario$Node[row_index]

post_probability <- get_exact_score_probability(

exact_score_distributions,

model_role = "Primary",

scenario = scenario_name,

target_node = target_node,

dose_sd = PRIMARY_DOSE_SD

)

post_counts <- sample_score_counts(

post_probability,

N_SIMULATED_SCORES

)

post_sample_moments <- sample_moments_from_counts(post_counts)

exact_post_moments <- distribution_moments(post_probability)

raw_p_value <- permutation_p_value_from_counts(

baseline_counts,

post_counts,

N_PERMUTATIONS,

PERMUTATION_BATCH_SIZE

)

output_rows[[row_index]] <- data.frame(

ModelRole = "Primary",

Model = primary_model$name,

Scenario = scenario_name,

DoseSD = PRIMARY_DOSE_SD,

Node = target_node,

Item = exact_scenario$Item[row_index],

Community = exact_scenario$Community[row_index],

ExactRank = exact_scenario$Rank[row_index],

SimulatedScoresPerCondition = N_SIMULATED_SCORES,

NumberOfPermutations = N_PERMUTATIONS,

BaselineSimulatedMean = unname(

baseline_sample_moments["Mean"]

),

PostSimulatedMean = unname(post_sample_moments["Mean"]),

SimulatedSignedMeanDifference = unname(

post_sample_moments["Mean"] -

baseline_sample_moments["Mean"]

),

ExactBaselineMean = primary_model$mean_sum_score,

ExactPostMean = unname(exact_post_moments["Mean"]),

ExactSignedMeanDifference =

unname(exact_post_moments["Mean"]) -

primary_model$mean_sum_score,

PermutationPValueTwoSided = raw_p_value,

stringsAsFactors = FALSE

)

}

output <- do.call(rbind, output_rows)

output$BonferroniPWithinDirection <- stats::p.adjust(

output$PermutationPValueTwoSided,

method = "bonferroni"

)

output$SignificantAfterBonferroni <-

output$BonferroniPWithinDirection < 0.05

output[order(output$ExactRank), ]

}

message("Running permutation validation for the primary 2-SD analysis.")

permutation_results <- rbind(

run_permutation_scenario("Aggravating", 1000L),

run_permutation_scenario("Alleviating", 2000L)

)

utils::write.csv(

permutation_results,

file.path(OUTPUT_DIR, "13_permutation_validation_primary_2SD.csv"),

row.names = FALSE

)

\# -----------------------------------------------------------------------------

\# 12. Repeated-simulation ranking stability of the primary 2-SD analysis

\# -----------------------------------------------------------------------------

run_stability_scenario <- function(scenario_name, seed_offset) {

set.seed(RANDOM_SEED + seed_offset)

baseline_probability <- get_exact_score_probability(

exact_score_distributions,

model_role = "Primary",

scenario = "Baseline",

target_node = "Original",

dose_sd = 0

)

baseline_count_matrix <- stats::rmultinom(

N_STABILITY_REPETITIONS,

N_SIMULATED_SCORES,

baseline_probability

)

baseline_means <- drop(

score_values %*% baseline_count_matrix

) / N_SIMULATED_SCORES

exact_scenario <- primary_2sd_results[

primary_2sd_results$Scenario == scenario_name,

]

exact_scenario <- exact_scenario[

match(node_labels, exact_scenario$Node),

]

post_means <- matrix(

NA_real_,

nrow = n_nodes,

ncol = N_STABILITY_REPETITIONS,

dimnames = list(

node_labels,

paste0("Rep", seq_len(N_STABILITY_REPETITIONS))

)

)

for (node_index in seq_len(n_nodes)) {

post_probability <- get_exact_score_probability(

exact_score_distributions,

model_role = "Primary",

scenario = scenario_name,

target_node = node_labels[node_index],

dose_sd = PRIMARY_DOSE_SD

)

post_count_matrix <- stats::rmultinom(

N_STABILITY_REPETITIONS,

N_SIMULATED_SCORES,

post_probability

)

post_means[node_index, ] <- drop(

score_values %*% post_count_matrix

) / N_SIMULATED_SCORES

}

effect_magnitude_matrix <- abs(

sweep(post_means, 2L, baseline_means, FUN = "-")

)

\# Sub-numerical random jitter avoids deterministic node-order bias when two

\# simulated discrete means are exactly equal.

tie_jitter <- matrix(

stats::runif(

length(effect_magnitude_matrix),

-1e-12,

1e-12

),

nrow = n_nodes,

ncol = N_STABILITY_REPETITIONS

)

rank_matrix <- apply(

effect_magnitude_matrix + tie_jitter,

2L,

function(values) rank(-values, ties.method = "first")

)

if (is.null(dim(rank_matrix))) {

rank_matrix <- matrix(rank_matrix, nrow = n_nodes)

}

rownames(rank_matrix) <- node_labels

summary_rows <- lapply(seq_len(n_nodes), function(node_index) {

node_effects <- effect_magnitude_matrix[node_index, ]

node_ranks <- rank_matrix[node_index, ]

current_exact <- exact_scenario[node_index, ]

data.frame(

ModelRole = "Primary",

Model = primary_model$name,

Scenario = scenario_name,

DoseSD = PRIMARY_DOSE_SD,

Node = node_labels[node_index],

Item = current_exact$Item,

Community = current_exact$Community,

ExactEffectMagnitude = current_exact$EffectMagnitude,

ExactRank = current_exact$Rank,

SimulatedScoresPerCondition = N_SIMULATED_SCORES,

NumberOfRepetitions = N_STABILITY_REPETITIONS,

MeanSimulatedEffectMagnitude = mean(node_effects),

SDSimulatedEffectMagnitude = stats::sd(node_effects),

MedianRank = stats::median(node_ranks),

RankQuantile2.5 = unname(stats::quantile(

node_ranks,

0.025,

names = FALSE

)),

RankQuantile97.5 = unname(stats::quantile(

node_ranks,

0.975,

names = FALSE

)),

Top1Frequency = mean(node_ranks == 1L),

Top5Frequency = mean(node_ranks <= TOP_K),

stringsAsFactors = FALSE

)

})

summary_table <- do.call(rbind, summary_rows)

summary_table <- summary_table[order(summary_table$ExactRank), ]

replicate_table <- data.frame(

Scenario = scenario_name,

Replicate = rep(

seq_len(N_STABILITY_REPETITIONS),

each = n_nodes

),

Node = rep(node_labels, times = N_STABILITY_REPETITIONS),

EffectMagnitude = as.vector(effect_magnitude_matrix),

Rank = as.integer(as.vector(rank_matrix)),

Top1 = as.integer(as.vector(rank_matrix)) == 1L,

Top5 = as.integer(as.vector(rank_matrix)) <= TOP_K,

stringsAsFactors = FALSE

)

list(summary = summary_table, replicates = replicate_table)

}

message("Running repeated-simulation ranking stability.")

stability_aggravating <- run_stability_scenario("Aggravating", 3000L)

stability_alleviating <- run_stability_scenario("Alleviating", 4000L)

stability_summary <- rbind(

stability_aggravating$summary,

stability_alleviating$summary

)

stability_replicates <- rbind(

stability_aggravating$replicates,

stability_alleviating$replicates

)

utils::write.csv(

stability_summary,

file.path(

OUTPUT_DIR,

"14_repeated_simulation_stability_primary_2SD.csv"

),

row.names = FALSE

)

utils::write.csv(

stability_replicates,

file.path(

OUTPUT_DIR,

"15_repeated_simulation_node_ranks_primary_2SD.csv"

),

row.names = FALSE

)

\# -----------------------------------------------------------------------------

\# 13. Moderation assessment

\# -----------------------------------------------------------------------------

moderation_all_tables <- NULL

moderation_significant_rows <- NULL

moderation_summary <- data.frame(

CandidateModeratorNodesTested = 0L,

Rule = MODERATION_RULE,

LambdaGamma = MODERATION_EBIC_GAMMA,

ResamplesPerModerator = MODERATION_RESAMPLES,

LowerQuantile = MODERATION_QUANTILES[1L],

UpperQuantile = MODERATION_QUANTILES[2L],

SignificantModeratorNodeCount = NA_integer_,

SignificantModerationRowCount = NA_integer_,

AnyResamplingIntervalExcludedZero = NA,

Status = "Not run",

stringsAsFactors = FALSE

)

if (isTRUE(RUN_MODERATION)) {

message(

"Running moderation assessment for ", n_nodes,

" candidate moderators."

)

moderation_checkpoint_directory <- file.path(

OUTPUT_DIR,

"moderation_checkpoints"

)

dir.create(

moderation_checkpoint_directory,

showWarnings = FALSE,

recursive = TRUE

)

input_signature <- paste(

unname(tools::md5sum(DATA_FILE)),

n_observations,

paste(colSums(binary_matrix), collapse = ":"),

sep = "|"

)

mgm_version <- as.character(utils::packageVersion("mgm"))

moderation_checkpoint_is_valid <- function(

checkpoint,

moderator_index

) {

is.list(checkpoint) &&

identical(checkpoint$schema_version, 1L) &&

identical(checkpoint$input_signature, input_signature) &&

identical(checkpoint$mgm_version, mgm_version) &&

identical(checkpoint$moderator_index, moderator_index) &&

identical(checkpoint$rule, MODERATION_RULE) &&

isTRUE(all.equal(

checkpoint$lambda_gamma,

MODERATION_EBIC_GAMMA

)) &&

identical(checkpoint$n_resamples, MODERATION_RESAMPLES) &&

isTRUE(all.equal(

checkpoint$quantiles,

MODERATION_QUANTILES

)) &&

is.data.frame(checkpoint$table) &&

all(c("Mod_qtl_low", "Mod_qtl_high") %in%

names(checkpoint$table))

}

run_one_moderator <- function(moderator_index) {

checkpoint_file <- file.path(

moderation_checkpoint_directory,

sprintf("moderator_%02d.rds", moderator_index)

)

checkpoint <- NULL

if (

isTRUE(REUSE_MODERATION_CHECKPOINTS) &&

file.exists(checkpoint_file)

) {

checkpoint <- tryCatch(

readRDS(checkpoint_file),

error = function(error_condition) NULL

)

}

if (moderation_checkpoint_is_valid(checkpoint, moderator_index)) {

message(

" moderator ", moderator_index, "/", n_nodes,

" (", node_labels[moderator_index], "): checkpoint reused."

)

return(checkpoint)

}

message(

" moderator ", moderator_index, "/", n_nodes,

" (", node_labels[moderator_index], ")."

)

set.seed(1314L + moderator_index)

moderated_model <- mgm::mgm(

data = binary_matrix,

type = rep("c", n_nodes),

level = rep(2L, n_nodes),

lambdaSel = "EBIC",

lambdaGam = MODERATION_EBIC_GAMMA,

ruleReg = MODERATION_RULE,

moderators = moderator_index,

scale = FALSE

)

resampling_result <- mgm::resample(

object = moderated_model,

data = binary_matrix,

nB = MODERATION_RESAMPLES,

quantiles = MODERATION_QUANTILES,

pbar = TRUE

)

raw_table <- as.data.frame(

mgm::plotRes(resampling_result, table = TRUE),

stringsAsFactors = FALSE

)

if (ncol(raw_table) < 8L) {

stop(

"mgm::plotRes returned an unexpected table for moderator ",

node_labels[moderator_index], "."

)

}

\# This column reduction follows the table returned by the mgm workflow

\# used in the reported analysis.

moderation_table <- raw_table[, -c(7L, 8L), drop = FALSE]

if (!all(c("Mod_qtl_low", "Mod_qtl_high") %in%

names(moderation_table))) {

stop(

"The moderation table lacks Mod_qtl_low or Mod_qtl_high for ",

node_labels[moderator_index], "."

)

}

significant_rows <- which(

(!is.na(moderation_table$Mod_qtl_low) &

moderation_table$Mod_qtl_low > 0) |

(!is.na(moderation_table$Mod_qtl_high) &

moderation_table$Mod_qtl_high < 0)

)

checkpoint <- list(

schema_version = 1L,

input_signature = input_signature,

mgm_version = mgm_version,

moderator_index = moderator_index,

rule = MODERATION_RULE,

lambda_gamma = MODERATION_EBIC_GAMMA,

n_resamples = MODERATION_RESAMPLES,

quantiles = MODERATION_QUANTILES,

table = moderation_table,

significant_rows = significant_rows

)

saveRDS(checkpoint, checkpoint_file)

checkpoint

}

moderation_results <- lapply(

seq_len(n_nodes),

run_one_moderator

)

moderation_all_tables <- do.call(

rbind,

lapply(seq_len(n_nodes), function(moderator_index) {

current_table <- moderation_results[[moderator_index]]$table

cbind(

data.frame(

ModeratorIndex = moderator_index,

ModeratorNode = node_labels[moderator_index],

ModeratorItem = node_names[moderator_index],

ModeratorCommunity = communities[moderator_index],

stringsAsFactors = FALSE

)[rep(1L, nrow(current_table)), , drop = FALSE],

current_table

)

})

)

rownames(moderation_all_tables) <- NULL

significant_parts <- list()

for (moderator_index in seq_len(n_nodes)) {

significant_indices <-

moderation_results[[moderator_index]]$significant_rows

if (length(significant_indices) == 0L) {

next

}

current_table <- moderation_results[[moderator_index]]$table[

significant_indices,

,

drop = FALSE

]

significant_parts[[length(significant_parts) + 1L]] <- cbind(

data.frame(

ModeratorIndex = moderator_index,

ModeratorNode = node_labels[moderator_index],

ModeratorItem = node_names[moderator_index],

ModeratorCommunity = communities[moderator_index],

stringsAsFactors = FALSE

)[rep(1L, nrow(current_table)), , drop = FALSE],

current_table

)

}

if (length(significant_parts) > 0L) {

moderation_significant_rows <- do.call(rbind, significant_parts)

rownames(moderation_significant_rows) <- NULL

} else {

moderation_significant_rows <-

moderation_all_tables[0, , drop = FALSE]

}

significant_moderator_count <- sum(vapply(

moderation_results,

function(result) length(result$significant_rows) > 0L,

logical(1)

))

moderation_summary <- data.frame(

CandidateModeratorNodesTested = n_nodes,

Rule = MODERATION_RULE,

LambdaGamma = MODERATION_EBIC_GAMMA,

ResamplesPerModerator = MODERATION_RESAMPLES,

LowerQuantile = MODERATION_QUANTILES[1L],

UpperQuantile = MODERATION_QUANTILES[2L],

SignificantModeratorNodeCount = significant_moderator_count,

SignificantModerationRowCount = nrow(moderation_significant_rows),

AnyResamplingIntervalExcludedZero =

nrow(moderation_significant_rows) > 0L,

Status = "Completed",

stringsAsFactors = FALSE

)

utils::write.csv(

moderation_all_tables,

file.path(OUTPUT_DIR, "16_moderation_all_resampling_tables.csv"),

row.names = FALSE

)

utils::write.csv(

moderation_significant_rows,

file.path(OUTPUT_DIR, "17_moderation_intervals_excluding_zero.csv"),

row.names = FALSE

)

}

utils::write.csv(

moderation_summary,

file.path(OUTPUT_DIR, "18_moderation_summary.csv"),

row.names = FALSE

)

\# -----------------------------------------------------------------------------

\# 14. Reproducibility records

\# -----------------------------------------------------------------------------

analysis_settings <- data.frame(

Setting = c(

"InputFile",

"NumberOfParticipants",

"ReportedSampleSize",

"RequiredItemColumns",

"NumberOfNodes",

"Dichotomisation",

"PrimaryModel",

"SensitivityModel",

"eLassoEBICGamma",

"eLassoRule",

"ExactStateCountPerModel",

"ExactBlockSize",

"PrimaryDoseSD",

"DoseSensitivitySD",

"SimulatedScoresPerCondition",

"PermutationsPerNode",

"RepeatedSimulationRepetitions",

"ModerationEBICGamma",

"ModerationRule",

"ModerationResamplesPerCandidate",

"RandomSeed"

),

Value = c(

basename(DATA_FILE),

n_observations,

REPORTED_SAMPLE_SIZE,

paste(node_labels, collapse = "; "),

n_nodes,

"0 versus 1-4",

primary_model$name,

sensitivity_model$name,

ELASSO_EBIC_GAMMA,

if (ELASSO_AND_RULE) "AND" else "OR",

2^n_nodes,

EXACT_BLOCK_SIZE,

PRIMARY_DOSE_SD,

paste(DOSES_SD, collapse = "; "),

N_SIMULATED_SCORES,

N_PERMUTATIONS,

N_STABILITY_REPETITIONS,

MODERATION_EBIC_GAMMA,

MODERATION_RULE,

MODERATION_RESAMPLES,

RANDOM_SEED

),

stringsAsFactors = FALSE

)

utils::write.csv(

analysis_settings,

file.path(OUTPUT_DIR, "00_analysis_settings.csv"),

row.names = FALSE

)

utils::write.csv(

item_dictionary,

file.path(OUTPUT_DIR, "00_MISSCARE_item_dictionary.csv"),

row.names = FALSE

)

capture.output(

sessionInfo(),

file = file.path(OUTPUT_DIR, "19_R_sessionInfo.txt")

)

saveRDS(

list(

settings = analysis_settings,

item_metadata = item_dictionary,

primary_model = primary_model,

sensitivity_model = sensitivity_model,

calibration_summary = calibration_summary,

calibration_node_probabilities = calibration_node_probabilities,

calibration_score_distributions = calibration_score_distributions,

exact_nira_results = exact_nira_results,

exact_score_distributions = exact_score_distributions,

parameterisation_comparison = parameterisation_comparison,

parameterisation_agreement = parameterisation_agreement,

cross_dose_robustness = cross_dose_robustness,

permutation_results = permutation_results,

stability_summary = stability_summary,

stability_replicates = stability_replicates,

moderation_summary = moderation_summary,

moderation_significant_rows = moderation_significant_rows

),

file.path(OUTPUT_DIR, "20_NIRA_reproducibility_objects.rds")

)

output_manifest <- data.frame(

File = sort(unique(c(

list.files(

OUTPUT_DIR,

recursive = TRUE,

full.names = FALSE

),

"21_output_manifest.csv"

))),

stringsAsFactors = FALSE

)

utils::write.csv(

output_manifest,

file.path(OUTPUT_DIR, "21_output_manifest.csv"),

row.names = FALSE

)

message(

"Analysis completed. Results were written to: ",

normalizePath(OUTPUT_DIR, mustWork = TRUE)

)
