# Supplementary material for: Missed Nursing Care Among Emergency and Critical Care Nurses: An Item‐Level Network Analysis With Computer‐Simulated Interventions
Source: J Nurs Manag. 2026 Sep 27;2026:5019343. doi: 10.1155/jonm/5019343 (PMC13616138; doi:10.1155/jonm/5019343)
Supplement: Supplementary file 1 [file JONM-2026-5019343-s001.zip › Supplementary materials8.24.docx]

**Supplementary materials**

| Supplementary Table S1. Full item wording, short labels, and content-based communities of missed nursing care items | | | |
| --- | --- | --- | --- |
| Node | Full item wording | Short label used in figures | Community |
| A1 | Changing patients’ bed linens in a timely manner | Bed linen care | Basic care |
| A2 | Turning patients every 2 hours | Turning every 2 hours | Basic care |
| A3 | Providing dietary guidance to patients | Dietary guidance | Planning |
| A4 | Providing mouth care | Mouth care | Basic care |
| A5 | Providing patient bathing/skin care | Bathing/skin care | Basic care |
| A6 | Assisting with toileting needs in a timely manner after request | Toileting assistance | Individual needs |
| A7 | Providing emotional support to patients and/or family members | Emotional support | Individual needs |
| A8 | Acting on PRN medication requests within 15 minutes | PRN medication | Individual needs |
| A9 | Administering medications within 30 minutes before or after the scheduled time | Timely medication administration | Individual needs |
| A10 | Responding to call lights in a timely manner | Call light response | Individual needs |
| A11 | Providing pressure ulcer, wound, or ostomy care | Pressure ulcer/wound/ostomy care | Basic care |
| A12 | Providing patient teaching about illness, tests, medications, and diagnostic studies | Health education | Planning |
| A13 | Providing rehabilitation and functional exercise guidance | Rehabilitation guidance | Planning |
| A14 | Performing focused reassessment and updating the nursing care plan according to patient condition | Focused reassessment/care-plan updating | Assessment |
| A15 | Providing IV/central line and drainage tube care and assessment according to hospital policy | IV/drainage tube care | Assessment |
| A16 | Performing timely risk assessment and anticipatory nursing care | Risk assessment | Assessment |
| A17 | Monitoring and documenting 24-hour intake and output as ordered | Intake/output monitoring | Assessment |
| A18 | Assessing vital signs as ordered | Vital sign monitoring | Assessment |
| A19 | Performing bedside blood glucose monitoring as ordered | Bedside glucose monitoring | Assessment |
| A20 | Completing full nursing documentation, including documentation of special treatments, procedures, and nursing outcome evaluation | Nursing documentation | Assessment |
| A21 | Reviewing medical records to fully understand assigned patients’ conditions | Medical record review | Assessment |
| A22 | Conducting ward rounds each shift according to graded nursing care standards | Ward rounds | Assessment |
| A23 | Providing discharge planning and discharge teaching | Discharge planning/teaching | Planning |
| A24 | Performing hand hygiene as indicated | Hand hygiene | Basic care |

| Supplementary Table S2. Original response distributions and dichotomized occurrence of missed nursing care items | | | | | | | |
| --- | --- | --- | --- | --- | --- | --- | --- |
| Item | Care activity | 0, n (%) | 1, n (%) | 2, n (%) | 3, n (%) | 4, n (%) | Any occurrence (1–4), n (%) |
| A1 | Bed linen care | 2,187 (65.26%) | 786 (23.46%) | 328 (9.79%) | 23 (0.69%) | 27 (0.81%) | 1,164 (34.74%) |
| A2 | Turning every 2 hours | 2,388 (71.26%) | 708 (21.13%) | 210 (6.27%) | 20 (0.60%) | 25 (0.75%) | 963 (28.74%) |
| A3 | Dietary guidance | 2,429 (72.49%) | 657 (19.61%) | 216 (6.45%) | 23 (0.69%) | 26 (0.78%) | 922 (27.51%) |
| A4 | Mouth care | 2,562 (76.45%) | 582 (17.37%) | 153 (4.57%) | 25 (0.75%) | 29 (0.87%) | 789 (23.55%) |
| A5 | Bathing/skin care | 2,447 (73.02%) | 663 (19.79%) | 180 (5.37%) | 33 (0.98%) | 28 (0.84%) | 904 (26.98%) |
| A6 | Toileting assistance | 2,698 (80.51%) | 492 (14.68%) | 120 (3.58%) | 13 (0.39%) | 28 (0.84%) | 653 (19.49%) |
| A7 | Emotional support | 2,438 (72.75%) | 695 (20.74%) | 167 (4.98%) | 22 (0.66%) | 29 (0.87%) | 913 (27.25%) |
| A8 | PRN medication | 2,696 (80.45%) | 500 (14.92%) | 117 (3.49%) | 14 (0.42%) | 24 (0.72%) | 655 (19.55%) |
| A9 | Timely medication administration | 2,783 (83.05%) | 452 (13.49%) | 81 (2.42%) | 9 (0.27%) | 26 (0.78%) | 568 (16.95%) |
| A10 | Call light response | 2,688 (80.21%) | 523 (15.61%) | 103 (3.07%) | 13 (0.39%) | 24 (0.72%) | 663 (19.79%) |
| A11 | Pressure ulcer/wound/ostomy care | 2,820 (84.15%) | 414 (12.35%) | 87 (2.60%) | 6 (0.18%) | 24 (0.72%) | 531 (15.85%) |
| A12 | Health education | 2,632 (78.54%) | 571 (17.04%) | 105 (3.13%) | 19 (0.57%) | 24 (0.72%) | 719 (21.46%) |
| A13 | Rehabilitation guidance | 2,603 (77.68%) | 595 (17.76%) | 110 (3.28%) | 16 (0.48%) | 27 (0.81%) | 748 (22.32%) |
| A14 | Focused reassessment/care-plan updating | 2,607 (77.80%) | 570 (17.01%) | 132 (3.94%) | 18 (0.54%) | 24 (0.72%) | 744 (22.20%) |
| A15 | IV/drainage tube care | 2,758 (82.30%) | 465 (13.88%) | 96 (2.86%) | 6 (0.18%) | 26 (0.78%) | 593 (17.70%) |
| A16 | Risk assessment | 2,645 (78.93%) | 579 (17.28%) | 86 (2.57%) | 15 (0.45%) | 26 (0.78%) | 706 (21.07%) |
| A17 | Intake/output monitoring | 2,960 (88.33%) | 291 (8.68%) | 67 (2.00%) | 7 (0.21%) | 26 (0.78%) | 391 (11.67%) |
| A18 | Vital sign monitoring | 2,986 (89.11%) | 275 (8.21%) | 58 (1.73%) | 6 (0.18%) | 26 (0.78%) | 365 (10.89%) |
| A19 | Bedside glucose monitoring | 2,954 (88.15%) | 309 (9.22%) | 57 (1.70%) | 7 (0.21%) | 24 (0.72%) | 397 (11.85%) |
| A20 | Nursing documentation | 2,806 (83.74%) | 436 (13.01%) | 78 (2.33%) | 7 (0.21%) | 24 (0.72%) | 545 (16.26%) |
| A21 | Medical record review | 2,593 (77.38%) | 599 (17.88%) | 126 (3.76%) | 9 (0.27%) | 24 (0.72%) | 758 (22.62%) |
| A22 | Ward rounds | 2,833 (84.54%) | 405 (12.09%) | 84 (2.51%) | 5 (0.15%) | 24 (0.72%) | 518 (15.46%) |
| A23 | Discharge planning/teaching | 2,742 (81.83%) | 484 (14.44%) | 90 (2.69%) | 11 (0.33%) | 24 (0.72%) | 609 (18.17%) |
| A24 | Hand hygiene | 2,642 (78.84%) | 552 (16.47%) | 119 (3.55%) | 14 (0.42%) | 24 (0.72%) | 709 (21.16%) |
| Note. 0 = Never missed; 1 = Rarely missed; 2 = Occasionally missed; 3 = Frequently missed; 4 = Always missed. Higher scores indicate more frequent missed nursing care. Any occurrence was defined as an original score of 1–4. Percentages were calculated using N = 3,351; there were no missing responses. | | | | | | | |

Supplementary Table S3. Exact calibration diagnostics for the calibrated original eLasso Ising model

| Panel A. Overall calibration and total-score distribution diagnostics | | | |
| --- | --- | --- | --- |
| Metric | Observed | Model-implied | Difference/summary |
| Mean sum score (0-24) | 4.932 | 4.932 | 0.000 |
| Mean activation proportion | 20.55% | 20.55% | 0.00 percentage points |
| Sum-score SD | 7.646 | 6.862 | 10.25% relative error |
| P(sum score = 0) | 48.52% | 25.22% | 23.31 percentage points |
| P(sum score = 24) | 7.49% | 2.16% | 5.33 percentage points |
| Pearson correlation across node probabilities | - | - | 0.969 |
| Mean absolute node-probability error | - | - | 0.026 |
| Maximum absolute node-probability error | - | - | 0.078 |
| Total variation distance | - | - | 0.304 |
| Kolmogorov-Smirnov distance | - | - | 0.233 |

| Panel B. Observed and model-implied activation probabilities by node | | | | | |
| --- | --- | --- | --- | --- | --- |
| Node | Community | Observed | Model-implied | Error (pp) | Absolute error (pp) |
| A1 | Basic care | 34.74% | 42.55% | +7.82 | 7.82 |
| A2 | Basic care | 28.74% | 33.14% | +4.40 | 4.40 |
| A3 | Planning | 27.51% | 31.43% | +3.92 | 3.92 |
| A4 | Basic care | 23.55% | 26.43% | +2.89 | 2.89 |
| A5 | Basic care | 26.98% | 30.90% | +3.93 | 3.93 |
| A6 | Individual needs | 19.49% | 22.29% | +2.80 | 2.80 |
| A7 | Individual needs | 27.25% | 32.06% | +4.81 | 4.81 |
| A8 | Individual needs | 19.55% | 18.07% | -1.48 | 1.48 |
| A9 | Individual needs | 16.95% | 13.06% | -3.89 | 3.89 |
| A10 | Individual needs | 19.79% | 19.54% | -0.25 | 0.25 |
| A11 | Basic care | 15.85% | 12.42% | -3.42 | 3.42 |
| A12 | Planning | 21.46% | 20.46% | -1.00 | 1.00 |
| A13 | Planning | 22.32% | 20.07% | -2.25 | 2.25 |
| A14 | Assessment | 22.20% | 20.67% | -1.53 | 1.53 |
| A15 | Assessment | 17.70% | 13.87% | -3.82 | 3.82 |
| A16 | Assessment | 21.07% | 19.58% | -1.49 | 1.49 |
| A17 | Assessment | 11.67% | 8.62% | -3.05 | 3.05 |
| A18 | Assessment | 10.89% | 10.29% | -0.60 | 0.60 |
| A19 | Assessment | 11.85% | 12.08% | +0.24 | 0.24 |
| A20 | Assessment | 16.26% | 15.75% | -0.51 | 0.51 |
| A21 | Assessment | 22.62% | 22.58% | -0.04 | 0.04 |
| A22 | Assessment | 15.46% | 11.34% | -4.12 | 4.12 |
| A23 | Planning | 18.17% | 14.04% | -4.13 | 4.13 |
| A24 | Basic care | 21.16% | 21.95% | +0.79 | 0.79 |

| Supplementary Table S4. Moderation-effect prerequisite assessment for the NIRA analysis | |
| --- | --- |
| Analysis specification/result | Value |
| Candidate moderator nodes assessed | 24 |
| Edge-selection rule | AND |
| EBIC tuning parameter | 0.25 |
| Nonparametric bootstrap samples per candidate moderator | 1000 |
| Bootstrap interval | 95% (2.5th-97.5th percentiles) |
| Moderator nodes with an interval excluding zero | 0 |
| Moderation-effect rows with an interval excluding zero | 0 |
| Note. Moderation effects were assessed using the AND rule, an EBIC tuning parameter of 0.25, and 1,000 nonparametric bootstrap samples per candidate moderator. NIRA = NodeIdentifyR algorithm. | |

Supplementary Table S5. Exact 2-SD simulated threshold-perturbation results for all 24 nodes in the calibrated original eLasso Ising model

| Panel A. Aggravating threshold perturbations | | | | | | |
| --- | --- | --- | --- | --- | --- | --- |
| Rank | Node | Community | Post-perturbation mean sum | Change in mean sum | Post-perturbation activation | Change (pp) |
| 1 | A15 | Assessment | 10.60 | +5.67 | 44.16% | +23.61 |
| 2 | A13 | Planning | 10.57 | +5.64 | 44.06% | +23.51 |
| 3 | A14 | Assessment | 10.46 | +5.53 | 43.57% | +23.02 |
| 4 | A12 | Planning | 10.41 | +5.48 | 43.37% | +22.82 |
| 5 | A23 | Planning | 10.33 | +5.40 | 43.05% | +22.50 |
| 6 | A16 | Assessment | 10.31 | +5.38 | 42.97% | +22.42 |
| 7 | A20 | Assessment | 10.31 | +5.38 | 42.95% | +22.40 |
| 8 | A11 | Basic care | 10.27 | +5.34 | 42.79% | +22.24 |
| 9 | A19 | Assessment | 10.22 | +5.29 | 42.59% | +22.04 |
| 10 | A18 | Assessment | 10.09 | +5.16 | 42.06% | +21.51 |
| 11 | A6 | Individual needs | 10.04 | +5.11 | 41.85% | +21.30 |
| 12 | A10 | Individual needs | 10.01 | +5.08 | 41.72% | +21.17 |
| 13 | A9 | Individual needs | 9.96 | +5.03 | 41.50% | +20.95 |
| 14 | A21 | Assessment | 9.91 | +4.98 | 41.29% | +20.74 |
| 15 | A4 | Basic care | 9.83 | +4.90 | 40.95% | +20.40 |
| 16 | A22 | Assessment | 9.78 | +4.85 | 40.76% | +20.21 |
| 17 | A17 | Assessment | 9.66 | +4.72 | 40.23% | +19.68 |
| 18 | A8 | Individual needs | 9.62 | +4.69 | 40.10% | +19.55 |
| 19 | A5 | Basic care | 9.55 | +4.62 | 39.81% | +19.26 |
| 20 | A7 | Individual needs | 9.51 | +4.58 | 39.61% | +19.06 |
| 21 | A3 | Planning | 9.32 | +4.38 | 38.82% | +18.27 |
| 22 | A24 | Basic care | 9.17 | +4.24 | 38.20% | +17.65 |
| 23 | A2 | Basic care | 9.07 | +4.14 | 37.79% | +17.24 |
| 24 | A1 | Basic care | 8.27 | +3.33 | 34.44% | +13.89 |

| Panel B. Alleviating threshold perturbations | | | | | | |
| --- | --- | --- | --- | --- | --- | --- |
| Rank | Node | Community | Post-perturbation mean sum | Change in mean sum | Post-perturbation activation | Change (pp) |
| 1 | A7 | Individual needs | 2.23 | -2.70 | 9.29% | -11.26 |
| 2 | A1 | Basic care | 2.24 | -2.69 | 9.34% | -11.21 |
| 3 | A5 | Basic care | 2.30 | -2.63 | 9.57% | -10.98 |
| 4 | A3 | Planning | 2.39 | -2.54 | 9.97% | -10.58 |
| 5 | A2 | Basic care | 2.41 | -2.52 | 10.03% | -10.52 |
| 6 | A4 | Basic care | 2.51 | -2.42 | 10.46% | -10.09 |
| 7 | A14 | Assessment | 2.68 | -2.25 | 11.17% | -9.38 |
| 8 | A13 | Planning | 2.68 | -2.25 | 11.17% | -9.38 |
| 9 | A12 | Planning | 2.72 | -2.22 | 11.32% | -9.23 |
| 10 | A6 | Individual needs | 2.73 | -2.20 | 11.37% | -9.18 |
| 11 | A21 | Assessment | 2.77 | -2.17 | 11.52% | -9.03 |
| 12 | A16 | Assessment | 2.82 | -2.11 | 11.76% | -8.79 |
| 13 | A10 | Individual needs | 2.94 | -1.99 | 12.26% | -8.29 |
| 14 | A20 | Assessment | 3.10 | -1.83 | 12.91% | -7.64 |
| 15 | A24 | Basic care | 3.13 | -1.80 | 13.03% | -7.52 |
| 16 | A15 | Assessment | 3.13 | -1.80 | 13.06% | -7.49 |
| 17 | A8 | Individual needs | 3.19 | -1.74 | 13.29% | -7.26 |
| 18 | A23 | Planning | 3.21 | -1.72 | 13.37% | -7.18 |
| 19 | A11 | Basic care | 3.33 | -1.60 | 13.89% | -6.66 |
| 20 | A19 | Assessment | 3.37 | -1.56 | 14.04% | -6.51 |
| 21 | A9 | Individual needs | 3.39 | -1.54 | 14.11% | -6.44 |
| 22 | A18 | Assessment | 3.52 | -1.41 | 14.65% | -5.90 |
| 23 | A22 | Assessment | 3.54 | -1.39 | 14.76% | -5.79 |
| 24 | A17 | Assessment | 3.73 | -1.20 | 15.53% | -5.02 |

| Supplementary Table S6. Permutation validation of the 2-SD NIRA threshold perturbations in the calibrated original eLasso model | | | | | | | | |
| --- | --- | --- | --- | --- | --- | --- | --- | --- |
| Scenario | Rank | Node | Post mean (SD) | 95% CI | Mean difference | Cohen's d | Raw p | Bonferroni p |
| Aggravating | 1 | A15 | 10.58 (9.37) | [10.32, 10.84] | +5.65 | 0.69 | 0.0002 | 0.0048 |
| Aggravating | 2 | A13 | 10.66 (8.90) | [10.42, 10.91] | +5.73 | 0.72 | 0.0002 | 0.0048 |
| Aggravating | 3 | A14 | 10.33 (8.90) | [10.08, 10.57] | +5.39 | 0.68 | 0.0002 | 0.0048 |
| Aggravating | 4 | A12 | 10.39 (8.85) | [10.14, 10.63] | +5.45 | 0.69 | 0.0002 | 0.0048 |
| Aggravating | 5 | A23 | 10.21 (9.30) | [9.96, 10.47] | +5.28 | 0.65 | 0.0002 | 0.0048 |
| Aggravating | 6 | A16 | 10.56 (9.01) | [10.31, 10.81] | +5.62 | 0.70 | 0.0002 | 0.0048 |
| Aggravating | 7 | A20 | 10.05 (9.23) | [9.79, 10.30] | +5.11 | 0.63 | 0.0002 | 0.0048 |
| Aggravating | 8 | A11 | 10.32 (9.40) | [10.06, 10.58] | +5.39 | 0.66 | 0.0002 | 0.0048 |
| Aggravating | 9 | A19 | 10.37 (9.56) | [10.11, 10.64] | +5.44 | 0.65 | 0.0002 | 0.0048 |
| Aggravating | 10 | A18 | 10.03 (9.65) | [9.76, 10.30] | +5.09 | 0.61 | 0.0002 | 0.0048 |
| Aggravating | 11 | A6 | 10.12 (8.58) | [9.88, 10.36] | +5.19 | 0.67 | 0.0002 | 0.0048 |
| Aggravating | 12 | A10 | 9.98 (8.96) | [9.73, 10.23] | +5.04 | 0.63 | 0.0002 | 0.0048 |
| Aggravating | 13 | A9 | 10.05 (9.27) | [9.79, 10.31] | +5.11 | 0.63 | 0.0002 | 0.0048 |
| Aggravating | 14 | A21 | 9.93 (8.88) | [9.68, 10.18] | +5.00 | 0.63 | 0.0002 | 0.0048 |
| Aggravating | 15 | A4 | 10.15 (8.44) | [9.91, 10.38] | +5.21 | 0.68 | 0.0002 | 0.0048 |
| Aggravating | 16 | A22 | 9.72 (9.53) | [9.45, 9.98] | +4.78 | 0.58 | 0.0002 | 0.0048 |
| Aggravating | 17 | A17 | 9.65 (9.68) | [9.38, 9.92] | +4.72 | 0.56 | 0.0002 | 0.0048 |
| Aggravating | 18 | A8 | 9.53 (8.85) | [9.29, 9.78] | +4.60 | 0.58 | 0.0002 | 0.0048 |
| Aggravating | 19 | A5 | 9.52 (8.24) | [9.29, 9.75] | +4.58 | 0.61 | 0.0002 | 0.0048 |
| Aggravating | 20 | A7 | 9.42 (8.23) | [9.19, 9.64] | +4.48 | 0.59 | 0.0002 | 0.0048 |
| Aggravating | 21 | A3 | 9.37 (8.19) | [9.14, 9.60] | +4.43 | 0.59 | 0.0002 | 0.0048 |
| Aggravating | 22 | A24 | 9.21 (8.83) | [8.97, 9.46] | +4.28 | 0.54 | 0.0002 | 0.0048 |
| Aggravating | 23 | A2 | 8.97 (8.16) | [8.74, 9.20] | +4.04 | 0.54 | 0.0002 | 0.0048 |
| Aggravating | 24 | A1 | 8.27 (7.80) | [8.05, 8.49] | +3.34 | 0.45 | 0.0002 | 0.0048 |
| Alleviating | 1 | A7 | 2.20 (3.78) | [2.09, 2.30] | -2.80 | -0.50 | 0.0002 | 0.0048 |
| Alleviating | 2 | A1 | 2.27 (4.39) | [2.15, 2.40] | -2.73 | -0.47 | 0.0002 | 0.0048 |
| Alleviating | 3 | A5 | 2.30 (4.03) | [2.19, 2.42] | -2.70 | -0.48 | 0.0002 | 0.0048 |
| Alleviating | 4 | A3 | 2.49 (4.38) | [2.36, 2.61] | -2.52 | -0.43 | 0.0002 | 0.0048 |
| Alleviating | 5 | A2 | 2.41 (4.31) | [2.29, 2.53] | -2.59 | -0.45 | 0.0002 | 0.0048 |
| Alleviating | 6 | A4 | 2.58 (4.26) | [2.46, 2.70] | -2.42 | -0.42 | 0.0002 | 0.0048 |
| Alleviating | 7 | A14 | 2.72 (4.11) | [2.61, 2.84] | -2.28 | -0.40 | 0.0002 | 0.0048 |
| Alleviating | 8 | A13 | 2.61 (4.01) | [2.50, 2.72] | -2.39 | -0.42 | 0.0002 | 0.0048 |
| Alleviating | 9 | A12 | 2.75 (4.30) | [2.63, 2.87] | -2.25 | -0.39 | 0.0002 | 0.0048 |
| Alleviating | 10 | A6 | 2.79 (4.51) | [2.67, 2.91] | -2.21 | -0.38 | 0.0002 | 0.0048 |
| Alleviating | 11 | A21 | 2.76 (4.23) | [2.64, 2.88] | -2.24 | -0.39 | 0.0002 | 0.0048 |
| Alleviating | 12 | A16 | 2.89 (4.35) | [2.77, 3.02] | -2.11 | -0.37 | 0.0002 | 0.0048 |
| Alleviating | 13 | A10 | 3.02 (4.61) | [2.89, 3.14] | -1.99 | -0.34 | 0.0002 | 0.0048 |
| Alleviating | 14 | A20 | 3.04 (4.39) | [2.92, 3.16] | -1.96 | -0.34 | 0.0002 | 0.0048 |
| Alleviating | 15 | A24 | 3.26 (4.92) | [3.12, 3.39] | -1.74 | -0.29 | 0.0002 | 0.0048 |
| Alleviating | 16 | A15 | 3.21 (4.70) | [3.08, 3.34] | -1.79 | -0.30 | 0.0002 | 0.0048 |
| Alleviating | 17 | A8 | 3.17 (4.82) | [3.04, 3.30] | -1.83 | -0.31 | 0.0002 | 0.0048 |
| Alleviating | 18 | A23 | 3.13 (4.59) | [3.00, 3.26] | -1.87 | -0.32 | 0.0002 | 0.0048 |
| Alleviating | 19 | A11 | 3.30 (4.87) | [3.16, 3.43] | -1.70 | -0.29 | 0.0002 | 0.0048 |
| Alleviating | 20 | A19 | 3.36 (4.86) | [3.22, 3.49] | -1.64 | -0.28 | 0.0002 | 0.0048 |
| Alleviating | 21 | A9 | 3.34 (4.93) | [3.20, 3.47] | -1.66 | -0.28 | 0.0002 | 0.0048 |
| Alleviating | 22 | A18 | 3.47 (5.04) | [3.33, 3.61] | -1.53 | -0.25 | 0.0002 | 0.0048 |
| Alleviating | 23 | A22 | 3.56 (5.16) | [3.42, 3.71] | -1.44 | -0.24 | 0.0002 | 0.0048 |
| Alleviating | 24 | A17 | 3.70 (5.34) | [3.55, 3.85] | -1.30 | -0.21 | 0.0002 | 0.0048 |

| Supplementary Table S7. Repeated-simulation stability of the 2-SD NIRA rankings in the calibrated original eLasso model | | | | | | | |
| --- | --- | --- | --- | --- | --- | --- | --- |
| Scenario | Exact rank | Node | Mean effect (SD) | 95% effect interval | Median rank [95% interval] | Top-1 frequency | Top-5 frequency |
| Aggravating | 1 | A15 | 5.67 (0.16) | [5.36, 5.99] | 2.0 [1.0, 6.0] | 45.5% | 97.3% |
| Aggravating | 2 | A13 | 5.65 (0.16) | [5.33, 5.95] | 2.0 [1.0, 6.0] | 35.1% | 96.7% |
| Aggravating | 3 | A14 | 5.53 (0.16) | [5.23, 5.84] | 3.0 [1.0, 9.0] | 9.7% | 79.8% |
| Aggravating | 4 | A12 | 5.49 (0.16) | [5.17, 5.80] | 4.0 [1.0, 9.0] | 6.0% | 68.1% |
| Aggravating | 5 | A23 | 5.41 (0.17) | [5.07, 5.73] | 6.0 [2.0, 11.0] | 1.7% | 43.6% |
| Aggravating | 6 | A16 | 5.39 (0.16) | [5.06, 5.71] | 6.0 [2.0, 11.0] | 0.9% | 39.4% |
| Aggravating | 7 | A20 | 5.37 (0.17) | [5.05, 5.68] | 7.0 [2.0, 12.0] | 0.8% | 33.1% |
| Aggravating | 8 | A11 | 5.34 (0.17) | [5.03, 5.68] | 7.0 [3.0, 12.0] | 0.2% | 23.1% |
| Aggravating | 9 | A19 | 5.29 (0.16) | [4.97, 5.61] | 8.0 [3.0, 13.0] | 0.1% | 15.3% |
| Aggravating | 10 | A18 | 5.17 (0.17) | [4.85, 5.51] | 10.0 [6.0, 15.0] | 0.0% | 1.8% |
| Aggravating | 11 | A6 | 5.12 (0.16) | [4.80, 5.44] | 11.0 [7.0, 15.0] | 0.0% | 1.0% |
| Aggravating | 12 | A10 | 5.09 (0.16) | [4.78, 5.40] | 12.0 [7.0, 16.0] | 0.0% | 0.4% |
| Aggravating | 13 | A9 | 5.03 (0.16) | [4.71, 5.33] | 13.0 [8.0, 17.0] | 0.0% | 0.4% |
| Aggravating | 14 | A21 | 4.98 (0.16) | [4.66, 5.29] | 14.0 [10.0, 17.0] | 0.0% | 0.0% |
| Aggravating | 15 | A4 | 4.90 (0.15) | [4.60, 5.20] | 15.0 [11.0, 18.0] | 0.0% | 0.0% |
| Aggravating | 16 | A22 | 4.86 (0.17) | [4.54, 5.19] | 16.0 [11.0, 19.0] | 0.0% | 0.0% |
| Aggravating | 17 | A17 | 4.73 (0.16) | [4.41, 5.04] | 17.0 [14.0, 20.0] | 0.0% | 0.0% |
| Aggravating | 18 | A8 | 4.69 (0.16) | [4.39, 4.99] | 18.0 [14.0, 21.0] | 0.0% | 0.0% |
| Aggravating | 19 | A5 | 4.63 (0.15) | [4.35, 4.92] | 19.0 [16.0, 21.0] | 0.0% | 0.0% |
| Aggravating | 20 | A7 | 4.58 (0.15) | [4.29, 4.85] | 19.0 [17.0, 21.0] | 0.0% | 0.0% |
| Aggravating | 21 | A3 | 4.40 (0.15) | [4.10, 4.70] | 21.0 [19.0, 23.0] | 0.0% | 0.0% |
| Aggravating | 22 | A24 | 4.25 (0.15) | [3.96, 4.57] | 22.0 [20.0, 23.0] | 0.0% | 0.0% |
| Aggravating | 23 | A2 | 4.14 (0.15) | [3.85, 4.44] | 23.0 [21.0, 23.0] | 0.0% | 0.0% |
| Aggravating | 24 | A1 | 3.35 (0.14) | [3.07, 3.63] | 24.0 [24.0, 24.0] | 0.0% | 0.0% |
| Alleviating | 1 | A7 | 2.70 (0.11) | [2.49, 2.92] | 2.0 [1.0, 3.0] | 46.9% | 100.0% |
| Alleviating | 2 | A1 | 2.69 (0.12) | [2.45, 2.92] | 2.0 [1.0, 4.0] | 41.5% | 100.0% |
| Alleviating | 3 | A5 | 2.64 (0.12) | [2.40, 2.86] | 3.0 [1.0, 5.0] | 10.7% | 99.9% |
| Alleviating | 4 | A3 | 2.53 (0.12) | [2.30, 2.76] | 4.0 [3.0, 6.0] | 0.4% | 93.0% |
| Alleviating | 5 | A2 | 2.52 (0.12) | [2.29, 2.76] | 5.0 [3.0, 6.0] | 0.4% | 90.8% |
| Alleviating | 6 | A4 | 2.42 (0.12) | [2.20, 2.65] | 6.0 [4.0, 7.0] | 0.1% | 16.3% |
| Alleviating | 7 | A14 | 2.25 (0.11) | [2.02, 2.47] | 8.0 [6.0, 11.0] | 0.0% | 0.0% |
| Alleviating | 8 | A13 | 2.25 (0.12) | [2.03, 2.47] | 8.0 [7.0, 11.0] | 0.0% | 0.0% |
| Alleviating | 9 | A12 | 2.21 (0.11) | [2.00, 2.44] | 9.0 [7.0, 12.0] | 0.0% | 0.0% |
| Alleviating | 10 | A6 | 2.20 (0.12) | [1.97, 2.44] | 10.0 [7.0, 12.0] | 0.0% | 0.0% |
| Alleviating | 11 | A21 | 2.17 (0.11) | [1.94, 2.40] | 11.0 [7.0, 12.0] | 0.0% | 0.0% |
| Alleviating | 12 | A16 | 2.11 (0.12) | [1.88, 2.33] | 12.0 [9.0, 13.0] | 0.0% | 0.0% |
| Alleviating | 13 | A10 | 1.99 (0.12) | [1.76, 2.22] | 13.0 [12.0, 14.0] | 0.0% | 0.0% |
| Alleviating | 14 | A20 | 1.83 (0.12) | [1.59, 2.07] | 15.0 [13.0, 18.0] | 0.0% | 0.0% |
| Alleviating | 15 | A24 | 1.81 (0.12) | [1.58, 2.04] | 15.0 [14.0, 18.0] | 0.0% | 0.0% |
| Alleviating | 16 | A15 | 1.80 (0.12) | [1.56, 2.02] | 15.0 [14.0, 18.0] | 0.0% | 0.0% |
| Alleviating | 17 | A8 | 1.74 (0.12) | [1.51, 1.98] | 17.0 [14.0, 19.0] | 0.0% | 0.0% |
| Alleviating | 18 | A23 | 1.72 (0.12) | [1.49, 1.95] | 17.0 [14.0, 19.0] | 0.0% | 0.0% |
| Alleviating | 19 | A11 | 1.60 (0.12) | [1.34, 1.84] | 19.0 [17.0, 22.0] | 0.0% | 0.0% |
| Alleviating | 20 | A19 | 1.56 (0.12) | [1.33, 1.79] | 20.0 [18.0, 22.0] | 0.0% | 0.0% |
| Alleviating | 21 | A9 | 1.54 (0.12) | [1.31, 1.78] | 21.0 [18.0, 22.0] | 0.0% | 0.0% |
| Alleviating | 22 | A18 | 1.41 (0.12) | [1.18, 1.65] | 22.0 [20.0, 23.0] | 0.0% | 0.0% |
| Alleviating | 23 | A22 | 1.39 (0.12) | [1.15, 1.63] | 23.0 [21.0, 24.0] | 0.0% | 0.0% |
| Alleviating | 24 | A17 | 1.20 (0.13) | [0.96, 1.45] | 24.0 [23.0, 24.0] | 0.0% | 0.0% |

Supplementary Table S8. Exact 2-SD simulated threshold-perturbation results for all 24 nodes in the calibrated post-selection sensitivity model

| Panel A. Aggravating threshold perturbations | | | | | | |
| --- | --- | --- | --- | --- | --- | --- |
| Rank | Node | Community | Post-perturbation mean sum | Change in mean sum | Post-perturbation activation | Change (pp) |
| 1 | A19 | Assessment | 14.84 | +9.91 | 61.84% | +41.29 |
| 2 | A18 | Assessment | 14.78 | +9.85 | 61.59% | +41.04 |
| 3 | A15 | Assessment | 14.75 | +9.82 | 61.46% | +40.91 |
| 4 | A17 | Assessment | 14.64 | +9.71 | 60.99% | +40.44 |
| 5 | A11 | Basic care | 14.56 | +9.62 | 60.65% | +40.10 |
| 6 | A23 | Planning | 14.54 | +9.61 | 60.59% | +40.04 |
| 7 | A20 | Assessment | 14.52 | +9.58 | 60.49% | +39.94 |
| 8 | A12 | Planning | 14.51 | +9.58 | 60.45% | +39.90 |
| 9 | A13 | Planning | 14.47 | +9.53 | 60.28% | +39.73 |
| 10 | A14 | Assessment | 14.44 | +9.51 | 60.17% | +39.62 |
| 11 | A9 | Individual needs | 14.41 | +9.48 | 60.03% | +39.48 |
| 12 | A16 | Assessment | 14.37 | +9.44 | 59.87% | +39.32 |
| 13 | A22 | Assessment | 14.33 | +9.40 | 59.71% | +39.16 |
| 14 | A10 | Individual needs | 14.10 | +9.17 | 58.75% | +38.20 |
| 15 | A6 | Individual needs | 14.04 | +9.11 | 58.52% | +37.97 |
| 16 | A4 | Basic care | 13.93 | +9.00 | 58.05% | +37.50 |
| 17 | A21 | Assessment | 13.88 | +8.95 | 57.85% | +37.30 |
| 18 | A8 | Individual needs | 13.87 | +8.94 | 57.80% | +37.25 |
| 19 | A5 | Basic care | 13.64 | +8.71 | 56.82% | +36.27 |
| 20 | A7 | Individual needs | 13.40 | +8.47 | 55.84% | +35.30 |
| 21 | A3 | Planning | 13.28 | +8.35 | 55.34% | +34.79 |
| 22 | A24 | Basic care | 13.16 | +8.22 | 54.82% | +34.27 |
| 23 | A2 | Basic care | 12.98 | +8.05 | 54.09% | +33.54 |
| 24 | A1 | Basic care | 11.71 | +6.78 | 48.80% | +28.25 |

| Panel B. Alleviating threshold perturbations | | | | | | |
| --- | --- | --- | --- | --- | --- | --- |
| Rank | Node | Community | Post-perturbation mean sum | Change in mean sum | Post-perturbation activation | Change (pp) |
| 1 | A7 | Individual needs | 1.34 | -3.59 | 5.58% | -14.97 |
| 2 | A1 | Basic care | 1.34 | -3.59 | 5.60% | -14.95 |
| 3 | A5 | Basic care | 1.37 | -3.56 | 5.72% | -14.83 |
| 4 | A2 | Basic care | 1.39 | -3.54 | 5.81% | -14.74 |
| 5 | A21 | Assessment | 1.42 | -3.52 | 5.90% | -14.65 |
| 6 | A4 | Basic care | 1.43 | -3.50 | 5.97% | -14.58 |
| 7 | A13 | Planning | 1.44 | -3.49 | 6.02% | -14.53 |
| 8 | A3 | Planning | 1.46 | -3.47 | 6.09% | -14.46 |
| 9 | A14 | Assessment | 1.47 | -3.46 | 6.12% | -14.43 |
| 10 | A16 | Assessment | 1.47 | -3.46 | 6.14% | -14.41 |
| 11 | A10 | Individual needs | 1.47 | -3.46 | 6.14% | -14.41 |
| 12 | A12 | Planning | 1.48 | -3.45 | 6.19% | -14.36 |
| 13 | A20 | Assessment | 1.50 | -3.43 | 6.25% | -14.30 |
| 14 | A23 | Planning | 1.54 | -3.39 | 6.41% | -14.14 |
| 15 | A19 | Assessment | 1.54 | -3.39 | 6.42% | -14.13 |
| 16 | A15 | Assessment | 1.57 | -3.36 | 6.53% | -14.02 |
| 17 | A18 | Assessment | 1.63 | -3.31 | 6.78% | -13.77 |
| 18 | A8 | Individual needs | 1.64 | -3.29 | 6.83% | -13.72 |
| 19 | A24 | Basic care | 1.64 | -3.29 | 6.85% | -13.70 |
| 20 | A9 | Individual needs | 1.65 | -3.28 | 6.88% | -13.67 |
| 21 | A22 | Assessment | 1.67 | -3.26 | 6.95% | -13.60 |
| 22 | A11 | Basic care | 1.68 | -3.25 | 7.02% | -13.53 |
| 23 | A6 | Individual needs | 1.74 | -3.20 | 7.23% | -13.32 |
| 24 | A17 | Assessment | 1.74 | -3.19 | 7.26% | -13.29 |

Supplementary Table S9. Agreement of NIRA effect rankings across calibrated models and threshold-perturbation doses

| Panel A. Overall ranking and top-five agreement | | | | | | |
| --- | --- | --- | --- | --- | --- | --- |
| Scenario | Dose (SD) | Spearman rho | Pearson r | Shared top-five, n | Jaccard index | Shared top-five nodes |
| Aggravating | 0.5 | 0.338 | 0.264 | 3 | 0.429 | A13; A14; A12 |
| Aggravating | 1.0 | 0.313 | 0.386 | 2 | 0.250 | A13; A12 |
| Aggravating | 1.5 | 0.581 | 0.704 | 2 | 0.250 | A12; A15 |
| Aggravating | 2.0 | 0.781 | 0.913 | 1 | 0.111 | A15 |
| Alleviating | 0.5 | 0.673 | 0.645 | 2 | 0.250 | A7; A5 |
| Alleviating | 1.0 | 0.773 | 0.755 | 2 | 0.250 | A7; A5 |
| Alleviating | 1.5 | 0.839 | 0.803 | 4 | 0.667 | A7; A5; A1; A2 |
| Alleviating | 2.0 | 0.851 | 0.826 | 4 | 0.667 | A7; A1; A5; A2 |

| Panel B. Nodes appearing in the top five of both models at one or more doses | | | | | |
| --- | --- | --- | --- | --- | --- |
| Scenario | Node | Community | Doses shared, n | Shared doses (SD) | Shared at all four doses |
| Aggravating | A12 | Planning | 3 | 0.5; 1; 1.5 | No |
| Aggravating | A13 | Planning | 2 | 0.5; 1 | No |
| Aggravating | A15 | Assessment | 2 | 1.5; 2 | No |
| Aggravating | A14 | Assessment | 1 | 0.5 | No |
| Alleviating | A5 | Basic care | 4 | 0.5; 1; 1.5; 2 | Yes |
| Alleviating | A7 | Individual needs | 4 | 0.5; 1; 1.5; 2 | Yes |
| Alleviating | A1 | Basic care | 2 | 1.5; 2 | No |
| Alleviating | A2 | Basic care | 2 | 1.5; 2 | No |

Fig. S1. Accuracy of the Ising network edge-weight estimates assessed using 1,000 nonparametric bootstrap samples. Red points indicate the sample estimates, black points indicate the bootstrap means, and grey bars indicate bootstrapped 95% confidence intervals.

Fig. S2. Case-dropping bootstrap stability of strength and expected influence in the Ising network based on 1,000 bootstrap samples. Lines represent the average correlations between centrality estimates obtained from case-dropped subsets and those obtained from the full sample; shaded areas indicate 95% confidence intervals.

Fig. S3. Agreement between observed and exact model-implied node activation probabilities for the calibrated original eLasso model (primary analysis) and the calibrated post-selection refit model (sensitivity analysis); dashed diagonal lines indicate perfect agreement.

Fig. S4. Observed and exact model-implied distributions of the number of activated nodes for the calibrated original eLasso model (primary analysis) and the calibrated post-selection refit model (sensitivity analysis).

Fig. S5. Permutation validation of model-implied mean sum scores following 2-SD node-threshold perturbations in the calibrated original eLasso model, based on 5,000 simulated observations per condition and 5,000 two-sided permutations; error bars indicate 95% normal-approximation confidence intervals, and asterisks indicate Bonferroni-adjusted permutation p values.

Fig. S6. Repeated-simulation stability of node rankings under 2-SD aggravating and alleviating threshold perturbations in the calibrated original eLasso model, based on 1,000 repetitions with 5,000 simulated observations per condition; symbols indicate top-1 and top-5 selection frequencies.

Fig. S7. Exact dose sensitivity of the five highest-ranked nodes across 0.5-, 1.0-, 1.5-, and 2.0-SD threshold perturbations in the calibrated original eLasso model (primary analysis) and the calibrated post-selection refit model (sensitivity analysis), shown separately for aggravating and alleviating directions.

Fig. S8. Gaussian graphical model of the 24 missed nursing care items estimated from the original 0–4 item scores using EBICglasso with Spearman correlations and a tuning parameter of 0.50.

Fig. S9. Bootstrapped 95% confidence intervals of the Gaussian graphical model edge weights based on 1,000 nonparametric bootstrap samples.

Fig. S10. Case-dropping bootstrap stability of strength and expected influence in the Gaussian graphical model based on 1,000 bootstrap samples.

Fig. S11. Standardized strength and expected influence estimates for the 24 missed nursing care items in the Gaussian graphical model.

Fig. S12. Bridge expected influence of the 24 missed nursing care items in the Gaussian graphical model.
